# Large-scale mitochondrial DNA analysis of native honey bee *Apis mellifera* populations reveals a new African subgroup private to the South West Indian Ocean islands

**DOI:** 10.1186/s12863-017-0520-8

**Published:** 2017-06-02

**Authors:** Maéva Angélique Techer, Johanna Clémencet, Christophe Simiand, Sookar Preeaduth, Hamza Abdou Azali, Bernard Reynaud, Delatte Hélène

**Affiliations:** 1 UMR PVBMT, Université de La Réunion, F-97715 Saint Denis cedex 9, La Réunion, France; 2 CIRAD, UMR PVBMT, 7 chemin de l’Irat, Ligne Paradis, 97410 Saint Pierre, La Réunion France; 3 Ministry of Agro Industry and Food Security, Entomology Division, Reduit, Republic of Mauritius; 4 Université des Comores, Route de la Corniche, BP 2585, Mkazi, Comoros; 5 Current Address: Okinawa Institute of Science and Technology Graduate University, Ecology and Evolution unit, 1919-1 Tancha Onna-son, Kunigami-gun, Okinawa, 904-0495 Japan

**Keywords:** COI-COII intergenic region, ND2, *A. M. unicolor*, Sequencing, Islands, Tropical, South West Indian ocean, Indigenous

## Abstract

**Background:**

The South West Indian Ocean (SWIO) archipelagos and Madagascar constitute a hotspot of biodiversity with a high rate of endemism. In this area, the endemic subspecies *A. m. unicolor* has been described in Madagascar. It belongs to the African lineage, one of the four described evolutionary lineages in honey bees. Despite a long beekeeping tradition and several recorded European introductions, few studies have been carried out on the diversity and proportion of honey bee subspecies.

In order to identify and define which evolutionary lineages and potential sub-lineages are present in the SWIO, the COI-COII intergenic region and the ND2 gene of the mtDNA were sequenced in honey bee colonies from three archipelagos. An extensive sampling (*n* = 1184 colonies) was done in the Mascarene (La Réunion, Mauritius, Rodrigues), Seychelles (Mahé, Praslin, La Digue) and Comoros (Grande Comore, Mohéli, Anjouan, Mayotte) archipelagos. Islands genetic diversity was compared to newly sampled populations from Madagascar, continental African and European populations.

**Results:**

African lineage haplotypes were found in all islands (except for Rodrigues). Madagascar, Comoros and Seychelles had 100% of A lineage, 95.5% in La Réunion and 56.1% in Mauritius. Among all African colonies detected in the SWIO, 98.1% (*n* = 633) of COI-COII haplotypes described the presence of the subspecies *A. M. unicolor*. Both genetic markers revealed i) a new private A_I_ mitochondrial group shared by the SWIO archipelagos and Madagascar distant from continental populations; ii) the private African haplotypes for each island suggested diversity radiation in the archipelagos; iii) the detection of the Comoros archipelago as a possible contact area between insular and continental African populations.

The exotic European C and M lineages were only detected in the Mascarene archipelago, but striking differences of proportion were observed among islands. Merely 4.6% of European colonies were found in La Réunion whereas Mauritius cumulated 44%. Here, among the 84 observed COI-COII haplotypes, 50 were newly described including 13 which were private to the SWIO archipelagos and Madagascar. Similarly, 24 of the 34 found ND2 haplotypes were novel which included six haplotypes particular to the SWIO populations.

**Conclusion:**

A new African subgroup was described in the SWIO region with mitochondrial genetic evidence that *A. m. unicolor* is the indigenous subspecies of the archipelagos surrounding Madagascar.

**Electronic supplementary material:**

The online version of this article (doi:10.1186/s12863-017-0520-8) contains supplementary material, which is available to authorized users.

## Background

The Western honey bee *Apis mellifera* is indigenous of Africa, Europe, Middle East and some regions in Asia but has spread worldwide due to beekeeping activities [1]. Three decades ago, morphometric approaches separated *A. mellifera* into 24 subspecies belonging to four distinct evolutionary branches: African lineage A, European lineages M and C, and Oriental lineage O [2]. Introduction of several molecular approaches (based on mtDNA, nuclear microsatellites, SNPs) [3–9] and geometric morphometric of wing shape slightly re-evaluated the micro taxonomy of *A. mellifera* [10]. Recent advent in genomic analysis has been a major key in honey bee intra-specific classification by keeping support of existing lineages [11] and at the same time enlarging the native range with the description of the subspecies *A. m. sinisxinyuan* [12].

The study of the singular evolution of the mitochondrial COI-COII intergenic region started in the early 1990’s [3, 13] which has since become a popular tool for discriminating evolutionary lineages in honey bee populations [14, 15]. The development of a PCR-RFLP test and specialized nomenclature has set up this marker to contain a transfer RNA gene for leucine (tRNA-leu), a non-coding insert and a partial sequence of the cytochrome oxidase unit II gene [16]. In *Apis mellifera*, the non-coding insert evolution helps to distinguish the African and European lineages mostly thanks to large variation of sequence size due to the absence/presence and repetition of P and Q units [15]. While the African A lineage is characterized by P_0_ or P_1_ units, the European M lineage contains only the P unit, whereas the European C lineage does not present any P unit [17]. The COI-COII intergenic region has been widely used to detect and track progressive importation of honey bee queens [18–22], phylogeographical studies in native areas [6, 23] and for the identification and understanding of secondary contact areas [24–26]. The sequences of COI-COII intergenic region from different lineages and subspecies has helped improve the knowledge and preserve indigenous honey bee populations. Earlier a disequilibrium in haplotype description among lineages was notable as European lineages were far more represented than the African lineage (91 M and 5 C vs 30 A haplotypes) [14]. The African lineage is yet an important reservoir of a genetic diversity, emphasized by the recent description of 113 mtDNA COI-COII haplotypes found in Iberian and North African honey bee colonies (742 individuals) [27].

The African continental scale analysis of the mitochondrial diversity using COI-COII describes four A sub-lineages [6]: the tropical A_I_ (widespread from Morocco to South Africa and South of Iberian peninsula), the A_II_ (Canary islands, Morocco, North of Africa from Algeria to Sicily), the Atlantic A_III_ (Iberian Peninsula and archipelagos) and the Yemenite Y (Ethiopia). The fifth African sub-lineage Z was described in Egypt and Syria using COI-COII marker and later confirmed by microsatellites analysis [9, 28]. Among the entire African lineage, 11 subspecies are currently recognized [29, 30]: *A. m. intermissa* and *A. m. sahariensis* in North Africa; *A. m. lamarckii*, *A. m. simensis* and *A. m. yemenitica* inhabiting North-East Africa; *A. m. adansonii* in the West and Central Africa; *A. m. scutellata* populating in Central and South Africa; *A. m. capensis* in South Africa, *A. m. monticola* in the mountains region of South-East Africa, *A. m. litorea* in the South-East of Africa and finally *A. m. unicolor* which is endemic to Madagascar. Morphometric [2, 10], microsatellites [31] and SNPs [7, 8] suggest that the insular *A. m. unicolor* subspecies is clearly differentiated from the continental subspecies in Africa. This analysis has been supported by the identification of 16 new COI-COII haplotypes from the African A_I_ sub-lineage in endemic *A. m. unicolor* populations from Madagascar [31].

Madagascar is a continental island which split from the African continent around 130 Myr ago and is currently located at 400 km from the East coast of Africa [32, 33]. In the South West of Indian Ocean region (SWIO), three archipelagos lie near Madagascar: Seychelles (~1100 km North East), Comoros (~300 km in the North of Mozambique Channel) and Mascarene archipelagos (~ 800 to 1500 km to the East). The granitic Seychelles archipelago is the oldest of the three and split from Madagascar around 88 Myr ago, followed by split from India around 64 Myr ago [33, 34]. Both Comoros and Mascarene archipelagos have a volcanic origin. Magmatic dating of the four islands of Comoros shows that it is 20 to 10 Myr old [35]. Mascarene archipelago (composed of three islands) on the other hand is relatively younger, La Réunion is the youngest (2.1 Myr) and still possess one of the most active volcanoes (intraplate hotspot) in the world [36, 37], while Mauritius is about 8.9 Myr old [38]. Within the archipelagos, all islands are separated by ocean barriers with a minimum of 11 km for the Seychelles archipelago (Praslin-La Digue) to more than 600 km for the Mascarene archipelago (Mauritius-Rodrigues) [38].

In all three archipelagos, *A. mellifera* is established and can be found in wild habitats. Modern and traditional beekeeping also exists in the Mascarene and Seychelles archipelagos. It is still unknown if the honey bees were present before human colonization (VII century in Comoros, XVII-XVIII centuries in the Mascarene and Seychelles archipelagos [39]). Importations of European *A. m. carnica*, *A. m. ligustica* and *A. m. mellifera* subspecies in the Mascarene and Seychelles archipelagos have been reported in historical records [1, 39–42]. Even though, importations have been forbidden in La Réunion since 1982 to prevent pathogen and parasite introduction. A recent study showed that in the Seychelles archipelago, all colonies (*n* = 121) harbored African lineage COI-COII sequences and 91.7% were similar to the sequences detected in Madagascar [43]. As for the Mascarene archipelago, the tropical African A_I_ sub-lineage was detected in La Réunion and Mauritius (*n* = 20 and 10 colonies collected in late 1990s, respectively) using the standard PCR-RFLP method on the COI-COII intergenic region [6]. The study on Rodrigues honey bee population revealed the exclusive presence of European C lineage (100%, *n* = 524) [44]. Unfortunately, genetic diversity of the honey bee populations in the Comoros archipelagos has not been investigated.

In order to have a complete understanding of the biogeography of *A. mellifera* in SWIO islands, we analysed the mitochondrial diversity of samples from the Mascarene (La Réunion, Mauritius and Rodrigues), Seychelles (Mahé, Praslin and La Digue) and Comoros archipelagos (Grande Comore, Mohéli, Anjouan and Mayotte). Outgroups from previously described continental African and European populations were used to get a better picture of the SWIO genetic diversity in a wider geographical context. Besides classical analysis of the mtDNA COI-COII intergenic region, we also investigated the ND2 gene diversity. This study aims to i) identify and describe the distribution of evolutionary lineages in the area, ii) assess the mitochondrial genetic diversity and structure of 11 insular honey bee populations and iii) understand the colonization and/or introduction patterns of *A. mellifera* in the SWIO archipelagos. Interestingly, indigenous and endemic species in these archipelagos have been previously demonstrated to share origins and/or evolutionary connections to Madagascar biodiversity with reported examples of species radiation in birds [45, 46], insects [47] and plants [48, 49]. We hypothesize that Madagascar could be the main population of origin of the honey bees from the SWIO archipelagos.

## Methods

### Sampling in the south west indian ocean (SWIO) islands

**Fig. 1:**
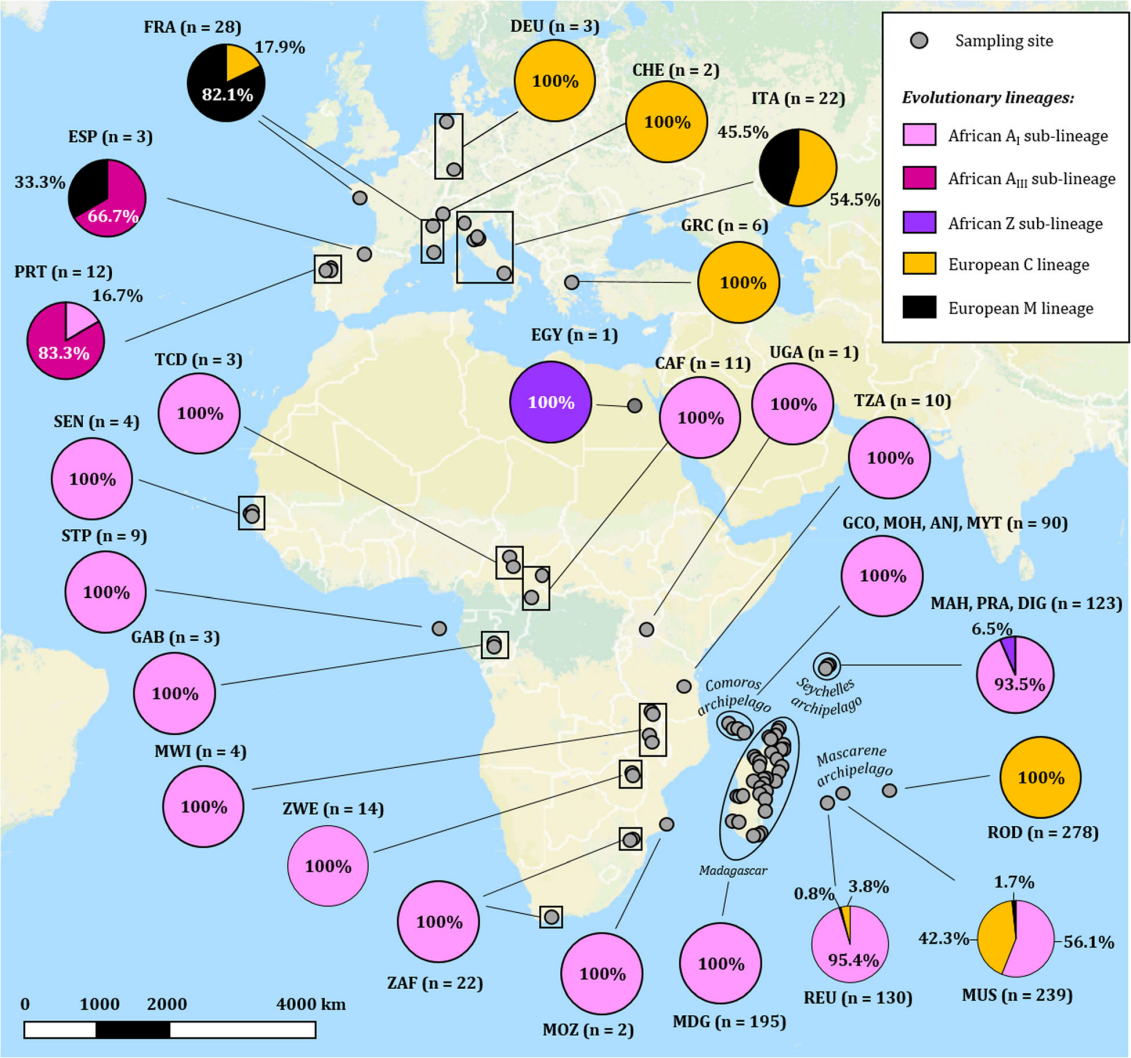
Evolutionary lineages transition within *Apis mellifera* native range using COI-COII region and distribution in the South West Indian Ocean archipelagos. Evolutionary lineages A, C and M and sub-lineages (A_I_, A_III_, Z) were determined by the sequencing of the mtDNA COI-COII intergenic region (*n* = 1184). Country and island codes are indicated near each frequency diagram with the associated number of sequence obtained. The map was constructed using QGIS and Open Landscape Layer from www.opencyclemap.org. *ANJ* Anjouan, *CAF* Central African Republic, *CHE* Switzerland, *COM* Comoros, *DEU* Germany, *DIG* La Digue, *EGY* Egypt, *ESP* Spain, *FRA* France, *GAB* Gabon, *GCO* Grande Comore, *GRC* Greece, *ITA* Italy, *MAH* Mahé, *MDG* Madagascar, *MOH* Mohéli, *MOZ* Mozambique, *MUS* Mauritius, *MYT* Mayotte, *MWI* Malawi, *PRA* Praslin, *PRT* Portugal, *REU* La Réunion, *ROD* Rodrigues, *SEN* Senegal, *STP* São Tomé Island, *SYC* Seychelles, *UGA* Uganda, *ZAF* South Africa, *ZWE* Zimbabwe and *TZA* Tanzania

A total of 1024 colonies were studied from the SWIO area including Madagascar (including 142 samples previously described using COI-COII intergenic region and microsatellites [31]); the Mascarene archipelago: La Réunion, Mauritius and Rodrigues (278 Rodrigues samples were previously described using COI-COII and microsatellites [44]); three islands of the Seychelles archipelago: Mahé, Praslin and La Digue (including 121 samples previously using COI-COII and microsatellites [43]) and the Comoros archipelago: Grande Comore, Mohéli, Anjouan and Mayotte (Table 1). One worker per colony was collected inside or at the entrance of the hive. Sampling locations are presented in Fig. 1 and for geographical coordinates details see Additional file 2: Table S1. The sampling sites in La Réunion (*n* = 83) and Mauritius (*n* = 24) were representative of the different habitats present on the islands (Additional file 1: Figure S1 and S2). All samples (except two feral colonies) were from managed colonies and came from 82 apiaries belonging to 64 beekeepers in La Réunion (one feral colony) and from 23 apiaries belonging to 15 beekeepers in Mauritius (one feral colony). In the Comoros archipelago as beekeeping is poorly developed, whenever possible honey bee foragers were collected every 5 km.

**Table 1:** Sampling location details for South West Indian Ocean islands, African and European populations by country

	Island Size	Code	*n*_site_	Date	*n*	*n*_COI-COII_	*n*_ND2_	Source
Madagascar	587,040 km^2^	MDG	4	1996-1998	17	17		b
			49	2011-2013	142	142	23	c
Madagascar (Ste Marie Island)	160 km^2^		1	2014	5	5	1	
Mascarene archipelago								
La Réunion	2512 km^2^	REU	83	2011- 2012	130	130	16	
Mauritius	1865 km^2^	MUS	24	2012	239	239	14	
Rodrigues	108 km^2^	ROD	20	2013	278	278	6	a
Seychelles archipelago								
Mahé	155 km^2^	MAH	17	2013	50	50	5	d
Praslin	38 km^2^	PRA	13	2013	45	45	4	d
			2	2015	2	2		
La Digue	10 km^2^	DIG	2	2013	26	26	4	d
Comoros archipelago								
Grande Comore	1148 km^2^	GCO	10	2014	29	29	8	
Mohéli	290 km^2^	MOH	3	2014	10	10	3	
Anjouan	424 km^2^	ANJ	11	2014	27	27	5	
Mayotte	376 km^2^	MYT	18	2013	24	24	4	
African populations								
Egypt		EGY	1	1997	1	1	1	b
Senegal		SEN	3	2015	4	4	4	
São Tomé Island	1001 km^2^	STP	1	1998	9	9	4	b
Chad		TCD	2	2015	3	3	1	
Central African Republic		CAF	5	2013	11	11	5	
Gabon		GAB	2	2014	3	3	3	
Uganda		UGA	1	2015	1	1	1	
Tanzania		TZA	1	2015	10	10	4	
Malawi		MWI	4	1995	4	4	3	b
Zimbabwe		ZWE	1	1995	5	5	3	b
			1	2014	9	9	6	
Mozambique		MOZ	1	2015	2	2	2	
South Africa		ZAF	1	2013	17	17	6	
			2	2015	5	5	3	
European populations								
Portugal		PRT	6	2013	12	12	4	
Spain		ESP	1	2013	3	3	1	
France		FRA	3	2013	28	28	13	
Switzerland		CHE	1	2013	2	2	1	
Germany		DEU	1	1998	2	2	2	b
			1	2013	1	1	1	
Italy		ITA	8	1997	22	22	8	b
Greece		GRC	1	2015	6	6	2	
					1184	1184	171	

Table details island size, map code (corresponding to Fig. 1), the number of sampling sites, date of the sampling, *n*: number of sampled honeybee colonies and *n*_COI-COII_ and *n*_ND2:_ number of individuals sequenced at the COI-COII intergenic region and ND2 gene, respectively. The reference source of samples from previous mtDNA genetic diversity description is indicated

^a^from Techer et al. (2015) [44]

^b^from the collection of INRA Toulouse (Franck [50])

^c^from Rasolofoarivao et al. [31]

^d^from Techer et al. (2016) [43]

### Sampling in the African and European populations

In Africa, a total of 84 colonies were sampled from 26 sites distributed in 12 countries: Egypt, Senegal, São Tomé Island, Chad, Central African Republic, Gabon, Uganda, Tanzania, Malawi, Zimbabwe, Mozambique and South Africa (Fig. 1, Table 1 and Additional file 2: Table S1). Samples from Egypt, São Tomé Island, Malawi, Zimbabwe (1995) were obtained from INRA Avignon [6, 50]. Based on geographical range, African sampling sites encompassed natural range of *A. m. adansonii*, *A. m. lamarckii*, *A. m. scutellata*, *A. m. capensis*, *A. m. monticola* and *A. m. litorea* subspecies.

In Europe, 76 colonies were sampled from 22 sites distributed in seven countries: Spain, Portugal, France, Switzerland, Germany, Italy and Greece. Samples from Germany (1998) and Italy were obtained from INRA Avignon [6, 50]. Based on geographical range, European sampling sites covered natural range of *A. m. mellifera*, *A. m. iberiensis*, *A. m. ligustica* and *A. m. cecropia* subspecies.

### DNA extraction

One worker per colony was used to analyse both mitochondrial markers. Total DNA was extracted from the legs. Legs were incubated in 400 μL of extraction buffer containing 0.1 M Tris HCl (pH 8.0), 80 mM EDTA, 100 mM NaCl, 200 mM sucrose, 2% SDS, 0.1 mg/ml proteinase K and H_2_O, overnight at 37 °C. Then extracts were centrifuged for 20 s at 17000 g, then 50 μl potassium acetate 8 M was added, followed by a centrifugation for 20 min at 17000 g. DNA was ethanol precipitated at ambient temperature, pelleted (30 min at 15322 g), rinsed in 95% ethanol, vacuum dried, and resuspended in 100 μL of Tris HCl 10 mM (pH = 8) and EDTA 1 mM. DNA concentration was then assayed by spectrophotometry (Nanodrop V8000) and diluted to 5 ng per μl for following analyses and stored at −20 °C.

### Amplification and sequencing of the mtDNA COI-COII intergenic and ND2 regions

All samples (*n* = 1184) were analysed for the COI-COII marker. PCR amplification of the intergenic COI-COII region was carried out using E2 (5′- GGCAGAATAAGTGCATTG -3′) and H2 (5′- CAATATCATTGATGACC -3′) primers following method described in Garnery et al. [16]. Then at least one individual per island or country and for each COI-COII haplotype detected was investigated at the ND2 region. ND2 gene was amplified using ILE (5′-TGATAAAAGAAATATTTTGA-3′) and L1 primers (5′-GAATCTAATTAATAAAAAA-3′) following the PCR method described in Arias et al. [4]. All PCR products were sent for sequencing to Macrogen©.

### Genetic diversity and phylogenetic analyses

Sequences were manually checked and aligned using Mega 5.1 [51]. All sequences with bad quality or ambiguous electrophoregrams were sequenced twice and completely removed if still unclear. Newly described haplotypes were submitted to GenBank NCBI database (Additional file 2: Table S1). The COI-COII haplotype description followed the nomenclature previously established for *A. mellifera* [14, 16]. This nomenclature is based on the structure of the sequence and localisation of *DraI* restriction sites. Evolutionary lineage for each individual was first check by size of the PCR fragment on gel and then confirmed by sequence blast on GenBank. All sequences started from the end 5′ of the E2 primer within the tRNA-leu and ended at the 3′ of “TTGTTTTCCATCATT” within the COII gene. Using ClustalW, alignments were performed on obtained sequences and other *A. mellifera* COI-COII sequences available on GenBank. Similarity among the COI-COII haplotypes was investigated using the goeBURST Minimal Spanning Tree from PHYLOVIZ [52] and POPART software [53]. Contrary to PHYLOVIZ which can deal with INDELs, COI-COII sequences were modified for the input format in POPART following the example of other studies on European populations [54]. A total of 21 GenBank reference sequences from the African lineage were implemented in the haplotype network. Regarding the size complexity of the COI-COII region, haplotype networks were computed independently for sequences with identical structure (i.e. P_0_Q, P_0_QQ, P_1_Q, Q, …). Population genetic statistics φ_PT_ and analysis of molecular variance were calculated using GenAlEx version 6.5 [55].

Haplotype network method was also applied for the ND2 partial gene sequences to assess the genetic diversity and structure. In addition, an Approximately-Maximum-Likelihood tree based on ND2 was computed using FastTree [56], using the Generalised Time Reversible (GTR) + CAT model and 1000 bootstrap replications. The phylogenetic tree was based on all ND2 haplotypes for each island or country and with at least one individual of each COI-COII haplotype. In addition, 49 sequences from other populations and subspecies were retrieved from GenBank and were incorporated in the analysis [4, 57, 58].

## Results

### COI-COII non-coding region

#### Evolutionary lineages and sub-lineages identification by COI-COII haplotypes

**Fig. 2:**
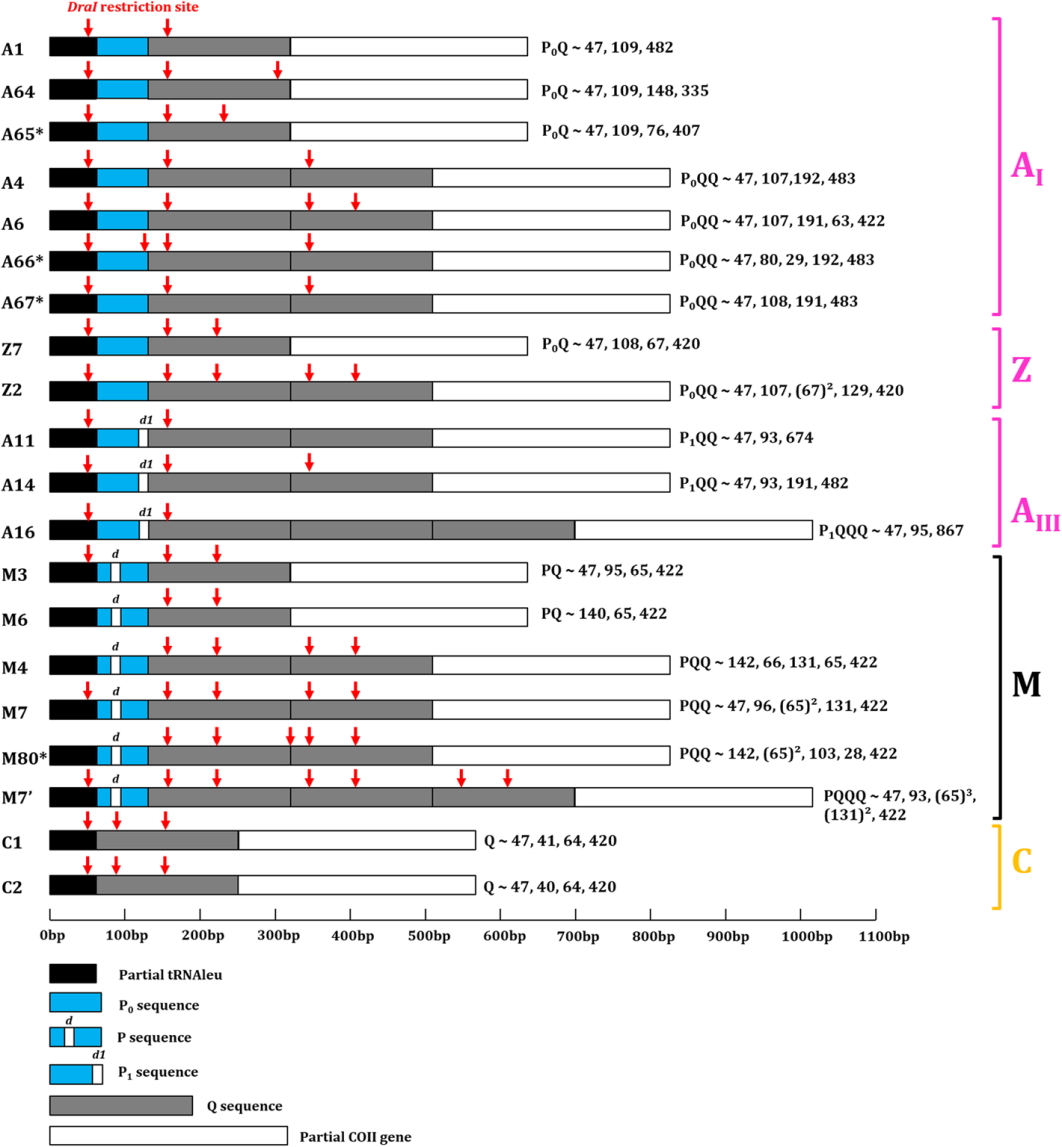
COI-COII honey bee sequences composition and *DraI* restriction patterns detected as a tool to discriminate evolutionary lineages and sub-lineages. For each restriction pattern, the composition in P (P_0_, P or P_1_) and Q units (from one to three units observed) is indicated. “d” and “d1” are characteristic deletions of the M lineage and Atlantic A lineage, respectively. Red arrows indicate restriction sites, numbers and restriction fragments sizes are also presented (~ indicates that size could slightly varied from a few bp due to insertion/deletion within sequence not modifying restriction sites). Profiles with * were newly described

DNA amplification and sequencing of the COI-COII intergenic region was successful for all analysed individuals (*n* = 1184). Three evolutionary lineages were detected [3]: the African A lineage (P_0_Q *n* = 647, P_0_QQ *n* = 73, P_1_QQ *n* = 9 and P_1_QQQ *n* = 3); the European C lineage (Q *n* = 413); and the European M lineage (PQ *n* = 5, PQQ *n* = 28 and PQQQ *n* = 6) (Additional file 1: Table S2). Three African sub-lineages A_I_ (*DraI* profiles A1, A4, A6, A64, A65, A66 and A67), A_III_ (*DraI* profiles A11, A14, A16) and Z (*DraI* profiles Z2, Z7), were identified from sequencing data coupled to *DraI* restriction profiles (Fig. 2).

**Fig. 3:**
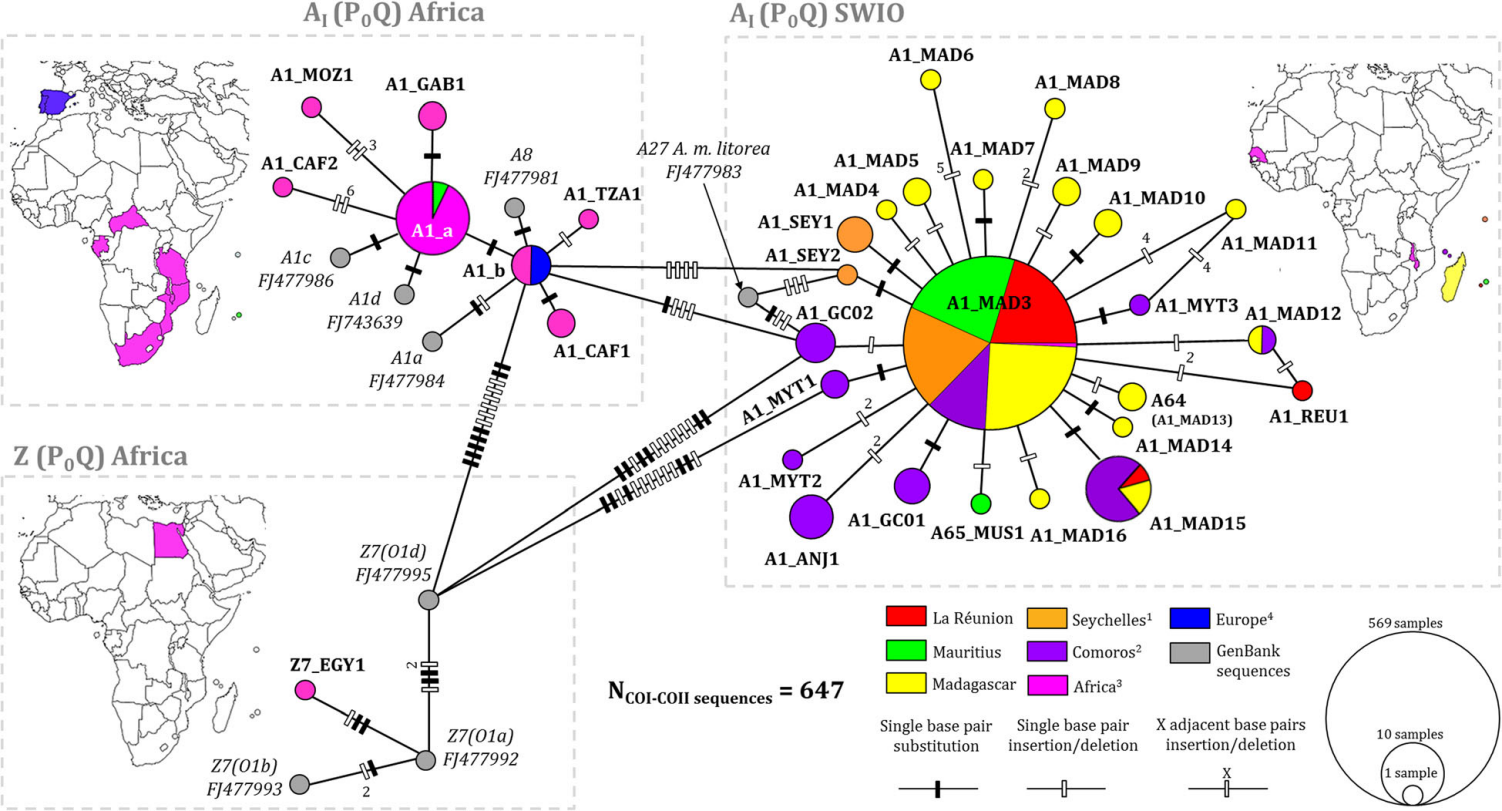
The sub-structure within 40 COI-COII P_0_Q derived haplotypes shows that the insular *A. mellifera* African A_I_ sub-lineage in the South West Indian Ocean differs from continental populations. The haplotype network is a minimal spanning tree based on 32 different haplotypes retrieved from sequencing 647 COI-COII of mtDNA from honey bees of the South West Indian Ocean islands, Africa, Europe and 8 African reference sequences from the GenBank. Maps near the sub-network indicate country/island from which sequences were obtained here using https://mapchart.net/ online tool. Seychelles^1^: Mahé, Praslin and La Digue. Comoros^2^: Grande Comore, Mohéli, Anjouan, and Mayotte. Africa^3^: Egypt, Senegal, Chad, Central African Republic, São Tomé, Gabon, Uganda, Tanzania, Malawi, Zimbabwe, Mozambique and South Africa. Europe^4^: Portugal, Spain, France, Germany, Switzerland, Italy and Greece

**Fig. 4:**
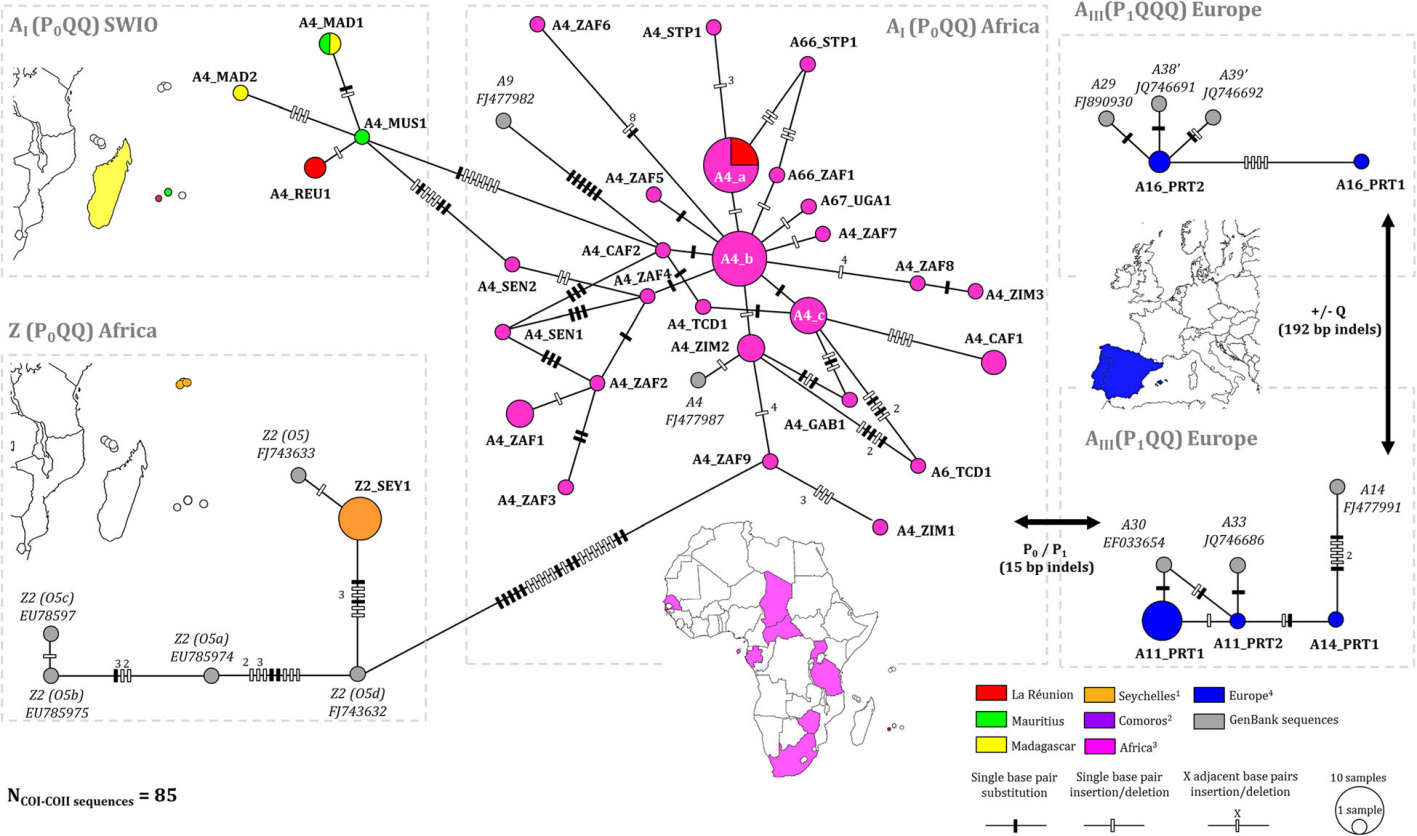
The sub-structure within 49 COI-COII P_0_QQ, P_1_QQ, and P_1_QQQ *A. mellifera* derived haplotypes shows the position of private insular haplotypes in regards to genetically similar continental populations. The haplotype network is a minimal spanning tree based on 36 different haplotypes retrieved from sequencing 85 COI-COII of mtDNA from honey bees of the South West Indian Ocean islands, Africa, Europe and 13 African reference sequences from the GenBank. Maps near the sub-network indicate country/island from which sequences were obtained here using https://mapchart.net/ online tool. Seychelles^1^: Mahé, Praslin and La Digue. Comoros^2^: Grande Comore, Mohéli, Anjouan, and Mayotte. Africa^3^: Egypt, Senegal, Chad, Central African Republic, São Tomé, Gabon, Uganda, Tanzania, Malawi, Zimbabwe, Mozambique and South Africa. Europe^4^: Portugal, Spain, France, Germany, Switzerland, Italy and Greece

Out of the 1184 individuals analysed, a total of 84 COI-COII haplotypes were verified in this survey including 50 newly described ones (Additional file 2: Table S1). The diversity of haplotypes per island and grouping by *DraI* classification is presented in Table 2 (detailed distribution of the diversity and occurrence in Additional file 1: Table S2). POPART and PHYLOVIZ outputs were highly similar, so only POPART networks are presented here in Figs. 3 and 4 for African haplotypes and in Additional file 1: Fig. S3 for European haplotypes. COI-COII haplotypes were split into 12 groups (consistent with the split of A, C and M lineages but also among African A_I_, A_III_, Z sub-lineages) regarding P and Q composition, *DraI* profile and geographical origin. Haplotypes associated with the three newly described African *DraI* profiles (Fig. 2) all clustered with others haplotypes from the A_I_ sub-lineage (Fig. 4). The newly described European haplotype (M80) clustered with M lineage haplotypes (Additional file 1: Figure S3).

**Table 2:** Number of COI-COII haplotypes classified by evolutionary lineages and *DraI* profile for each island or continental population

		La Réunion	Mauritius	Rodrigues	Mahé	Praslin	La Digue	Grande Comore	Anjouan	Mohéli	Mayotte	Madagascar	Egypt	Senegal	Chad	CAF	São Tomé	Gabon	Uganda	Tanzania	Malawi	Zimbabwe	Mozambique	South Africa	Portugal	Spain	France	Germany	Switzerland	Italy	Greece
A_I_	A1	3	2		3	1	1	4	3	1	5	13		1		4		1		3	3		3	1	1						
	A4	2	2									2		2	2	4	2	1		1		5		10							
	A6														1																
	A64											1																			
	A65		1																												
	A66																1							1							
	A67																		1												
	Z2					1	1																								
Z	Z7												1																		
	A11																								2	1					
A_III_	A14																								1						
	A16																								2						
	M3																													1	SS
	M4	1																									2				
M	M6																										2				
	M7		1																											1	
	M7’																									1	1			1	
	M80																										1				
C	C1		1	2																							1		1	1	
	C2	4	1	1																							1	3			1
	*Total haplotype*	10	8	3	3	2	2	4	3	1	5	16	1	3	3	8	3	2	1	4	2	5	3	12	6	2	8	3	1	4	1
	*Private A*	2	2		2	1^a^	2	1		3	12	1	2	2	4	2	2	1	1		3	1	9	4							
	*Private M*		1																							4			2		
	*Private C*			1																							1				

^a^For Praslin and La Digue, one haplotype was considered private to both islands (Z2_SEY1)

#### Distribution of the evolutionary A, C, M lineages and A_I_, A_III_ and Z sub-lineages

Geographical distribution of the evolutionary lineages and sub-lineages according to COI-COII sequencing is presented in Fig. 1. In the SWIO region, as previously described [31], newly sampled colonies from Madagascar were all from the African A_I_ sub-lineage (Table 2). Colonies from the Comoros archipelago were 100% from the African A_I_ sub-lineage and exhibited nine haplotypes exclusively associated with A1 *DraI* profile. In the Seychelles archipelago, all colonies (previous sampling 2013 and new sampling 2015) from Mahé had A_I_ sub-lineage sequences (A1_MAD3, A1_SEY1, A1_SEY2) whereas 91.5% of A_I_ (A1_MAD3) and 8.5% of Z sub-lineages (Z2_SEY1) were found in Praslin and similarly in La Digue (91.7% of A_I_, 8.3% of Z) [43]. Concerning La Réunion and Mauritius, three evolutionary lineages were detected including the African A and both European C and M, however in different proportions (Fig. 1). La Réunion was characterized by 95.4% of the colonies from the African A_I_ sub-lineage (A1_MAD3, A1_REU1, A4_REU1), 3.8% from European C lineage (C2_a, C2_b, C2_c, C2_d) and 0.8% from European M lineage (M4_a). In the Mauritius sampling, African and European lineages were equally detected with 56.1% of the colonies belonging to the A_I_ sub-lineage (A1_MAD3, A4_MUS1, AD_MAD1, A65_MUS1), 42.3% to the C lineage (C1_A, C2_a) and only 1.7% to the M lineage (M7_MUS1).

All sampled colonies from Africa (*n* = 84) exhibited COI-COII haplotypes described in the African A_I_ sub-lineage with the exception of Egypt for which the only colony sampled was from the Z sub-lineage (Z7_EGY1). In European populations, as previously described, colonies in Portugal and Spain possessed haplotypes from the Atlantic African A_III_ sub-lineage [6, 59, 60]. Expectations of finding M lineage haplotypes in Spain, France and Italy from earliest description were fulfilled, while C lineage haplotypes were detected in France, Germany, Switzerland, Italy and Greece colonies [17, 61, 62].

#### African COI-COII haplotype diversity: A_I_, A_III_ and Z sub-lineages

Among the 31 haplotypes of the African lineage detected in the SWIO samples, 29 were only found in the SWIO populations (Additional file 1: Table S2). The COI-COII haplotype A1_MAD3, first described in native *A. m. unicolor* populations [31] was the most represented in the present sampling on all islands 1) in Madagascar at 87.2%, 2) in the Seychelles archipelago at 92% in Mahé, 91.5% in Praslin and 84.6% in La Digue, 3) in the Comoros archipelago at 100% in Mohéli, 79.2% in Mayotte, 77.8% in Anjouan and 51.7% in Grande Comore, and 4) in the Mascarene archipelago at 89.2% in La Réunion, 54.4% in Mauritius and with the exception of Rodrigues. This particular A1_MAD3 haplotype was spread all over sampling sites from La Réunion and Mauritius islands (Additional file 1: Figures S1, S2). For the first time, A1_MAD3 was also described in two geographically distant sites in continental Africa in Senegal (*n* = 2) and Malawi (*n* = 2).

Almost all SWIO islands presented at least one private African COI-COII haplotype highly similar (≤ 99%) to A1_MAD3 including Madagascar (*n*_*hap*_ = 11), Mahé (*n*_*hap*_ = 2), Grande Comore (*n*_*hap*_ = 2), Mayotte (*n*_*hap*_ = 3), Anjouan (*n*_*hap*_ = 1), Mauritius (*n*_*hap*_ = 1) and La Réunion (*n*_*hap*_ = 1) (Fig. 3). In the SWIO area, the highest number of different haplotypes were detected in Madagascar (*n*_*hap*_ = 16, *n* = 164 colonies) but regarding sample size Grande Comore (*n*_*hap*_ = 4, *n* = 29 colonies) and Mayotte (*n*_*hap*_ = 5, *n* = 24 colonies) were ranked first (Table 2).

Haplotypes from the A_I_ sub-lineage found on SWIO islands differed from African continental haplotypes by between 3 and >10 mutations (Fig. 3). The A27 reference sequence [FJ477983] collected from the *A. m. litorea* native range was the closest continental haplotype to SWIO private haplotypes and more particularly to A1_GCO2 and A1_SEY2. Highest φ_PT_ significant values appeared between the African P_0_Q colonies from the SWIO and African P_0_Q colonies from Central African Republic and Tanzania (both *n* > 5) (see Additional file 1: Table S3). Concerning the A_I_ (P_0_Q) SWIO group (*n* = 24), a star-like pattern appeared with A1_MAD3 as the central node from which radiated highly similar sequences (99% of identity, up to five adjacent INDELs). Within these African P_0_Q SWIO populations, φ_PT_ values were significant and indicated moderate differentiation between Grande Comore (φ_PT_ max = 0.286, *p* = 0.001) and the other islands, and same applies for Anjouan (φ_PT_ max = 0.280, *p* = 0.001) (Additional file 1: Table S3). Haplotype A1_a included in A_I_ (P_0_Q) African group was observed in one site of Mauritius and also in six sites from five African countries. Results from blastn revealed that A1_a was already described in Argentine and USA Africanized populations [GenBank: JQ582437, JQ582438, GU326335] [63] and A1_b in *A. m. iberiensis* from the Iberian Peninsula [GenBank: FJ477985] [6] and in Brazil and Uruguay [GenBank: EF033649] [64].

Similarly, the A_I_ (P_0_QQ) SWIO group (haplotypes A4_MAD1, A4_MAD2, A4_REU1 and A4_MUS1) clearly differed from the A_I_ (P_0_QQ) group found in continental African populations with at least eight mutations steps. The most diverse group with 26 haplotypes was the continental African A_I_ (P_0_QQ) group (Fig. 4). From this continental group, only the haplotype A4_a was retrieved in SWIO islands and exclusively in La Réunion (*n* = 3 colonies). The haplotype node A4_a was already found in Argentina [GenBank: JQ582439] and has been detected here in Central African Republic, São Tomé Island and Tanzania (*A. m. adansonii, A. m. scutellata* and *A. m. litorea* range). The haplotype A4_b, previously found in Brazil and Uruguay [GenBank: EF033650] [64], was the central node of A_I_ (P_0_QQ) Africa group and has been observed in samples from South Africa and Zimbabwe.

Concerning the two African A_III_ sub-lineage groups, the five haplotypes detected in *A. m. iberiensis* natural range (Portugal and Spain) were all newly described (A11_PRT1, A11_PRT2, A14_PRT1, A16_PRT1 and A16_PRT2). Both Z sub-lineages groups were clearly differentiated from SWIO and others continental African sub-lineages (Figs. 3 and 4). Finally, in accordance with COI-COII haplotype nomenclature, A1_MAD13 [31] was renamed A64.

#### European COI-COII haplotypes: C and M lineages

Three COI-COII sequences groups from the M lineage were detected based on the P/Q unit composition (Additional file 1: Figure S3). Among the nine haplotypes detected in our sampling (*n* = 34 colonies) five were described for the first time (Additional file 2: Table S1). PQ (M3_a, M6_FRA1 and M6_FRA2) and PQQQ (M7’_a) were only detected in European populations. M3_a was previously described in USA colonies [GenBank: GQ85621] [65]. M4_a was detected in one colony from La Réunion, 11 colonies from France and was already described in protected areas of *A. m. mellifera* from Belgium and France [GenBank: KF274625, KF274636] [66]. Likewise, M4_b haplotype was already described in native protected areas of *A. m. mellifera* from Belgium, Netherlands and Norway [GenBank: KF274627, KF274628] [66]. In Mauritius, M lineage was detected in only four colonies having M7_MUS1 haplotype. M7_MUS1 haplotype was 99% identical (one deletion) to M7_a detected in four sites of Italy in which *A. m. ligustica* occurs. This haplotype has formerly been observed in North American populations [GenBank: FJ743636 which might have been misnamed as M3 instead of M7] [63].

Among the C lineage, seven haplotypes were detected in our sampling, four of them were detected both in Europe and in the Mascarene archipelago, one was only found in Europe (C2_DEU1) and two (C1_ROD1, C2_a) were only detected in the Mascarene archipelago. C1_a have been previously observed in Rodrigues [44] and was also detected in 20 colonies from Mauritius. C1_a haplotype is considered as characteristic of *A. m. ligustica* populations and was already found in Italy [6]. C2_a was present in all islands of the Mascarene archipelago, and was previously observed in Argentina [GenBank: JQ582431], in Canary Islands [GenBank: JF723978, HQ199228.1] in Turkey [GenBank: FJ037776] [67]. Out of the 24 sites in Mauritius, two presented high proportions of C2_a sequences and were apiaries from the same beekeeper (Additional file 1: Fig. S2). Finally C2_b, c and d haplotypes were solely detected in La Réunion and European populations (Additional file 1: Figure S3) and were identical to haplotypes observed in Turkey [GenBank: FJ037777] [67], in *A. m. carnica* colonies from Serbia [GenBank: JQ977702] [68] and Romania [GenBank: HQ270149] [69].

### ND2 haplotypes distribution and phylogeny

**Fig. 5:**
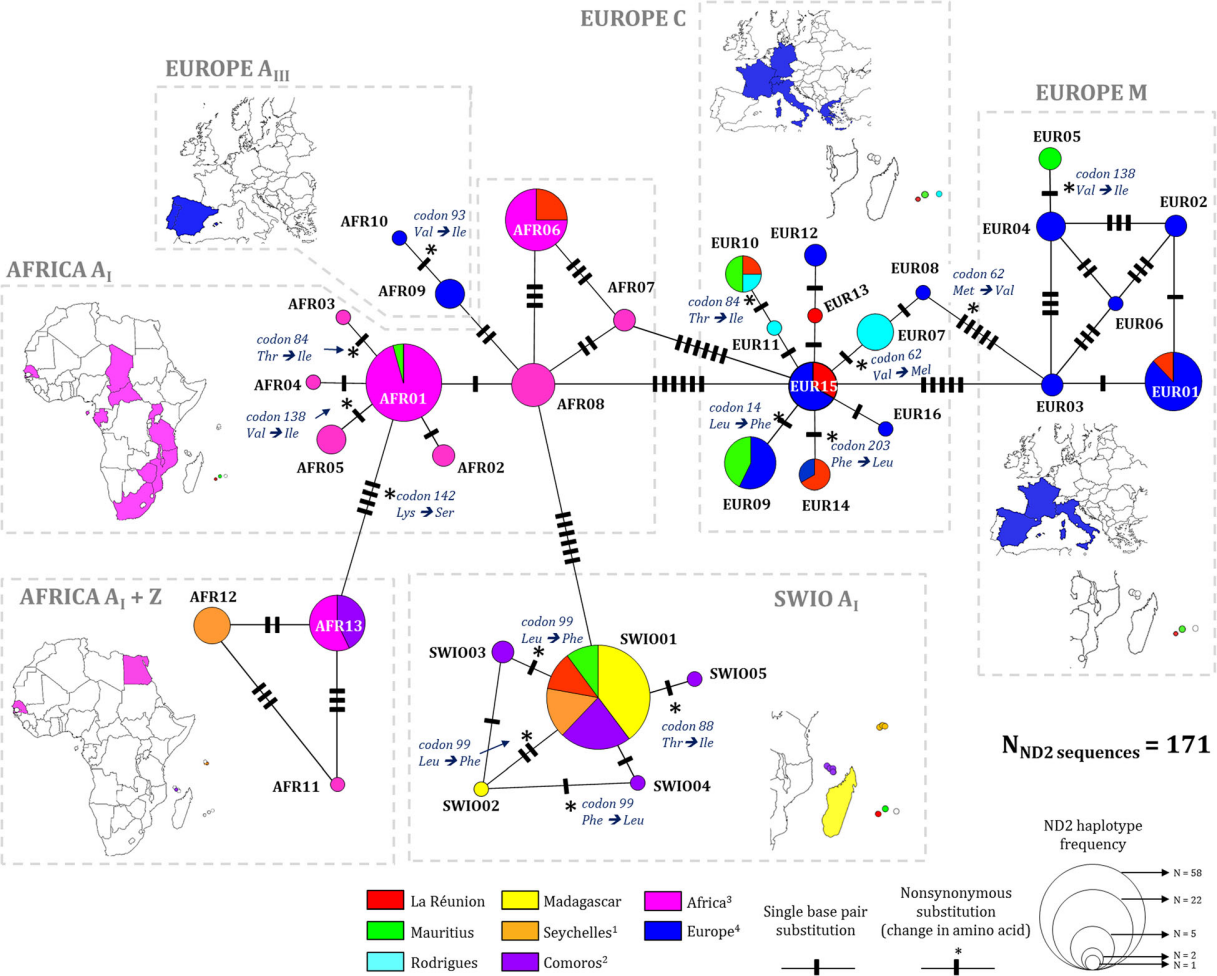
The South West Indian Ocean *A. mellifera* African ND2 haplotypes form a star-like group and diverge from the continental African and European lineages populations. The haplotype network is a minimal spanning tree based on 34 different haplotypes retrieved from sequencing 171 ND2 mtDNA from honey bees of the South West Indian Ocean (SWIO) islands, Africa, Europe. Maps near the sub-network indicate country/island from which sequences were obtained here using https://mapchart.net/ online tool. Seychelles^1^: Mahé, Praslin and La Digue. Comoros^2^: Grande Comore, Anjouan, Mohéli, and Mayotte. Africa^3^: Egypt, Senegal, Chad, Central African Republic, São Tomé, Gabon, Uganda, Tanzania, Malawi, Zimbabwe, Mozambique and South Africa. Europe^4^: Portugal, Spain, France, Germany, Switzerland, Italy and Greece

A total of 34 haplotypes of the ND2 region were obtained from the 171 individuals sequenced in this survey (Additional file 2: Table S1). Details about their distribution in sampled populations are given in Additional file 1: Table S4. Results from sequence alignment and blast showed that 23 haplotypes were newly described ones. The ND2 haplotype network showed the presence of six groups according to their geographical distribution, and grouping was also consistent with the split of COI-COII evolutionary lineages and sub-lineages (Fig. 5). The ND2 haplotypes found in continental African populations and SWIO islands were divided into three groups 1) Africa A_I_ group (*n* = 8); 2) SWIO A_I_ (*n* = 5); and 3) a mixed sub-lineages group Africa A_I_ + Z (*n* = 3). The SWIO A_I_ group was different from at least six synonymous substitutions from the continental African A_I_ group. On the other hand, the African A_I_ + Z group was distant from at least four mutation steps to continental African A_I_ group, including one non-synonymous substitution common to AFR11, 12 and 13.

Similar to the COI-COII non-coding region, African ND2 haplotypes to the SWIO formed a star-like pattern and were all newly described and private (Fig. 5). The SWIO01 haplotype was common to all archipelagos and islands (63.4%, *n* = 59 colonies) except for Rodrigues and Mohéli. Madagascar possessed the private SWIO02 sequence highly similar to SWIO01 (≤ 99%). Relatively high genetic diversity was found in the Comoros archipelago with three private ND2 haplotypes including SWIO03 in Grande Comore, SWIO04 in Mayotte and SWIO05 in Anjouan. Concerning haplotypes found in continental African populations, only AFR01 was detected in one colony of Mauritius and AFR06 in two colonies of La Réunion.

Mascarene archipelago was the sole SWIO island system to share ND2 haplotypes with European populations and even possess private one (Fig. 5). Out of the 10 ND2 haplotypes of the C group, seven were detected in the Mascarenes, including EUR07 and EUR11 private to Rodrigues and EUR13 private to La Réunion. Concerning the M group, EUR01 was detected in La Réunion, EUR 05 was private to Mauritius.

#### ND2 and COI-COII haplotypes comparison

**Fig. 6:**
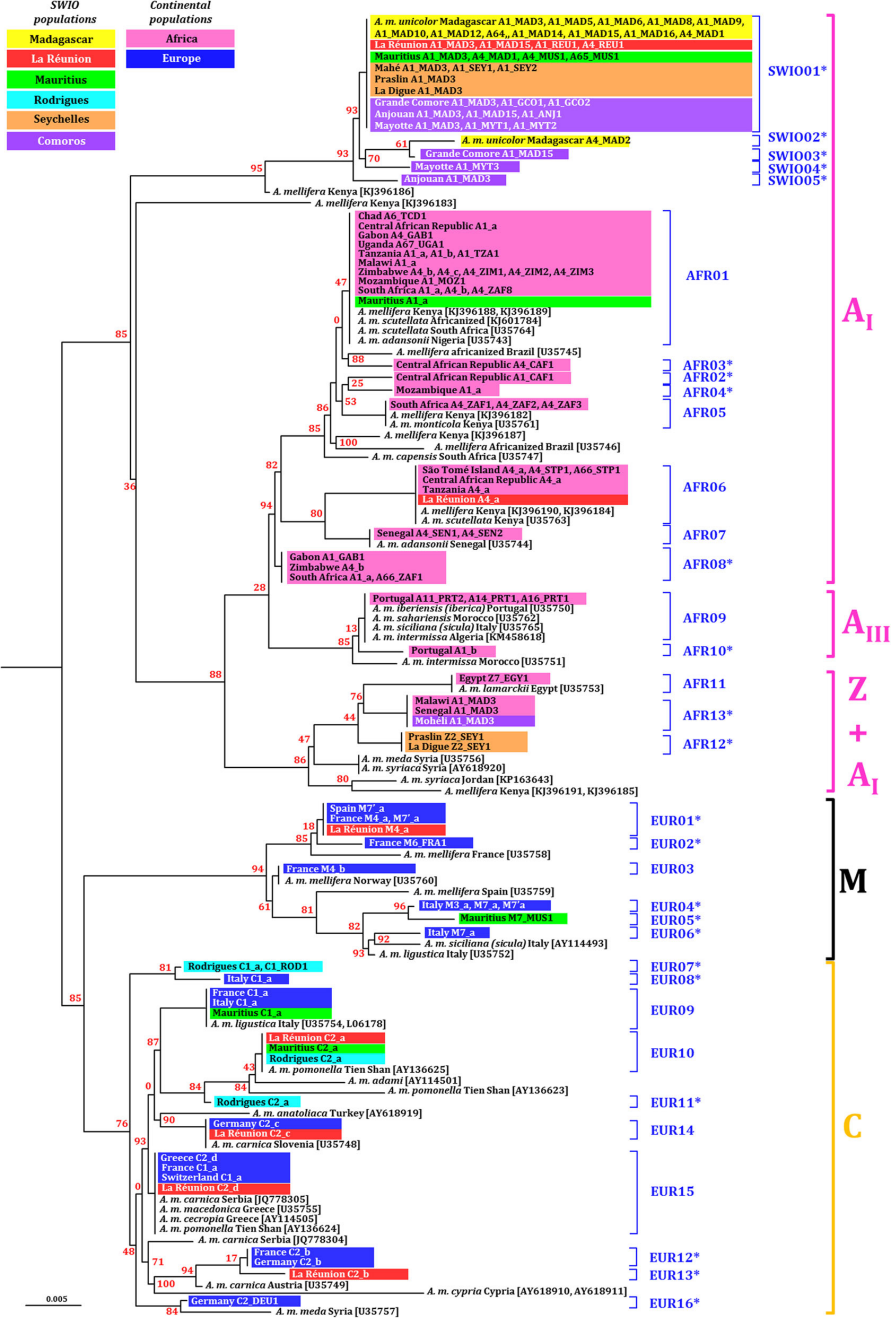
Phylogenetic relations among ND2 haplotypes linked to COI-COII haplotypes and geographical origins support a South West Indian Ocean *A. mellifera* African sub-group. The tree is an Approximately-Maximum-Likelihood phylogenetic tree based on ND2 haplotype and using GTR + CAT model. The tree was based on the 34 obtained ND2 sequences, including 49 references sequences from GenBank (in brackets). For each ND2 haplotype, the origin of the sample is indicated (South West Indian Ocean islands, African or European countries) and highlighted by a color. Additionally, the observed associated COI-COII haplotype is also specified for each island or country taxon. Only one individual of each ND2–COI-COII and location are represented in the tree for better visibility (see also Additional file 1: Table S4). Bootstraps are indicated in red near the node

A total of 83 combinations among ND2 and COI-COII haplotypes were detected in the dataset (Additional file 1: Table S4). The SWIO01 ND2 haplotype was associated with 23 COI-COII haplotypes which was the largest observed number. However, six COI-COII haplotypes showed an association with several ND2 haplotypes, such as the case of A1_MAD3 detected in individuals bearing SWIO01, SWIO05 or AFR13. As for haplotype networks (ND2 and COI-COII), AML tree showed a structure according to geographic localization (continental Africa, SWIO and Europe) and evolutionary lineages/sub-lineages classification. An African ND2 mitochondrial group (AFR01-13 and SWIO01-05) was verified and was exclusively associated to African COI-COII haplotypes (Fig. 6). The second main group gathered European ND2 haplotypes (EUR01-16) also entirely linked to European COI-COII either from C or M lineage.

Within the African group, an A_I_ cluster private to Madagascar and archipelagos (SWIO01-05, 85% of bootstraps) was visibly detached from continental African A_I_ haplotypes (AFR01-08, 13). No African subspecies reference sequences other than the newly described sequences of *A. m. unicolor* clustered to that insular African group. Two individuals from La Réunion were more closely related to both *A. m. scutellata* and *A. m. adansonii* (80% bootstraps) reference sequences. This observation was consistent with the COI-COII haplotype as A4_a was not detected in *A. m. unicolor* populations from Madagascar. The same observation was made for one individual in Mauritius (A1_a) clustered in the *A. m. scutellata/A. m. adansonii* AFR01 group. Out of the private SWIO cluster another particular African cluster appeared containing AFR11-13 ND2 sequences and both A_I_ and Z COI-COII sequences. Branches from this Z + A_I_ cluster, gathered sequences from Middle East, North Africa subspecies *A. m. syriaca*, *A. m. lamarckii*, *A. m. meda,* the Z2_SEY1 individuals from Seychelles archipelago as well as individuals from Mohéli, Senegal and Malawi having the main SWIO COI-COII haplotype A1_MAD3. Observed polymorphism within the ND2 marker was not high enough to discriminate African subspecies because sequences from up to four subspecies could be clustered together.

In parallel, the European M group was well distinguished from the other European C group (also including O lineage subspecies sequences). The M4_a haplotype (COI-COII) individual from La Réunion was closely related to *A. m. mellifera* sequences whereas M7_MUS haplotype (COI-COII) individuals from neighbouring Mauritius clustered with *A. m. ligustica* sequences. With the exception of the EUR10 cluster, none of the identified C2-like haplotypes (COI-COII) from Mascarene clustered together (see Fig. 6, EUR 11 to EUR16).

#### Levels of divergence among continental and insular African mitochondrial groups

The haplotype divergence was calculated only for the ND2 region as the COI-COII estimation is more complicated due to large insertion/deletions that could results from one or several mutations steps. The divergence magnitude among and within ND2 groups are presented in Table 3. Nucleotide divergence among sequences from the SWIO A_I_ insular group and the A_I_ continental Africa mitochondrial group was high (1.44 ± 0.48). When compared to all other groups, average divergence levels of SWIO A_I_ were higher than observed divergence between older described African sub-lineages A_I_ and A_III_ (0.64 ± 0.32) and comparable to divergence between evolutionary lineages such as A and C (1.28 ± 0.32 to 1.61 ± 0.48).

**Table 3:** Means of ND2 sequence divergence percentage (± SD) within (in bold) and between mitochondrial groups of honey bees defined according to haplotype network

		A lineage				C lineage	M lineage
	ND2 group	SWIO A_I_	AFRICA A_I_	AFRICA A_I_ + Z	EUROPE A_III_	EUROPE C	EUROPE M
A lineage	SWIO A_I_	**0.32 (±0.16)**					
	AFRICA A_I_	1.44 (±0.48)	**0.48 (±0.16)**				
	AFRICA A_I_ + Z	1.93 (±0.48)	0.96 (±0.32)	**0.48 (±0.16)**			
	EUROPE A_III_	1.61 (±0.48)	0.64 (±0.32)	0.96 (±0.32)	**0.16 (±0.16)**		
C lineage	EUROPE C	1.44 (±0.48)	1.28 (±0.32)	1.61 (±0.48)	1.61 (±0.48)	**0.32 (±0.16)**	
M lineage	EUROPE M	1.93 (±0.48)	1.77 (±0.48)	1.93 (±0.48)	1.77 (±0.48)	1.28 (±0.32)	**0.48 (±0.16)**

## Discussion

The COI-COII intergenic region has been widely used to detect evolutionary lineages and study the genetic diversity of honey bee populations [15]. On the other hand, ND2 gene sequences are highly conserved and offer less resolution to discriminate among subspecies [4, 58, 70]. Sequencing of the COI-COII non-coding (*n* = 1184) and the ND2 coding (*n* = 171) regions complement each other. They showed that the African lineage, and especially *A. m. unicolor,* were predominant in the Mascarene (La Réunion, Mauritius), the Comoros (Grande Comore, Anjouan, Mayotte) and in the Seychelles (Mahé, Praslin, La Digue) archipelagos. A new genetic and geographic structure was detected between the South West Indian Ocean (SWIO) to the well documented continental populations. For the first time, a novel mitochondrial African subgroup was reported outside of *A. mellifera* native continental range, as it was unique to Madagascar and nearby archipelagos. It also supported the hypothesis of ancient colonization of honey bees in the islands. At the same time, this study questions earlier description of *A. m. unicolor* being solely endemic to Madagascar and suggesting that the subspecies is most probably also indigenous of nearby archipelagos. Interestingly, genetic signals from some exotic European honey bees in the Mascarene archipelago were also observed in this study.

### Which evolutionary lineages are present in the honey bee populations from the SWIO?

Three out of the four evolutionary lineages defined for *A. mellifera* including A, C and M lineages were detected in the SWIO islands as confirmed by both mtDNA markers. The African lineage naturally encompasses the African continent, the Iberian peninsula and nearby islands such as Cape Verde, Azores and Canary archipelagos and most importantly Madagascar [6]. Due to geographical proximity of Mascarene, Seychelles and Comoros archipelagos to Madagascar it was not surprising to find that most of the sampled colonies in the area belonged to the African lineage (61.9%, *n*_COI-COII_ = 634 in Madagascar and archipelagos) of which 98.1% possessed *A. M. unicolor*-like haplotypes (*n*_COI-COII_ = 622). Nevertheless, contrasting patterns of lineage assembling was observed among islands.

In Madagascar, genetic assessment of colonies collected from 1996 to 2014 period showed that only the tropical African A_I_ sub-lineage was settled and widespread throughout the island [6, 31]. Until now, only the Cape Verde [6, 71] and Madagascar were the only known cases of insular honey bee populations exclusively constituted of A_I_ sub-lineage. But here, the Comoros archipelago (Grande Comore, Mohéli, Anjouan and Mayotte) was revealed to be another case of islands completely populated by African A_I_ sub-lineage. As a comparison in the same region, the Seychelles archipelago showed mainly African A_I_ sub-lineage colonies, with a few African Z sub-lineage colonies detected in Praslin and La Digue [43]. The Z sub-lineage distribution was thought to cover North and East African continental populations (Somalia, Egypt) [6] and in the Middle East (Syria, Iraq, Lebanon) [28], but here phylogenetic relations based on the ND2 region indicates that the range of this sub-lineage becomes broader.

Contrary to the two other archipelagos from the SWIO, the Mascarene archipelago was the sole where the African A_I_ sub-lineage cohabits with both European C and M lineages. The most extreme case was the honey bee population from Rodrigues which was previously described as exclusively belonging to the C lineage using COI-COII [44]. This assertion is now confirmed by the ND2 marker. La Réunion had a more dominant African lineage (95.4%) as compared to European lineages (3.8% of C and 0.8% of M) whereas Mauritius had similar proportions (African 56.1% vs. European lineages 42.3% of C and 1.7% of M). Nevertheless, the results for Mauritius should be considered with caution as African lineage frequency might have been underestimated. A previous study has seen biased results due to low sampling errors (La Réunion *n* = 20 and Mauritius *n* = 30, one site each) [6] and high proportion of C lineage in Mauritius may be caused by the sur-representation of one beekeeper. Indeed, two apiaries with the highest frequencies of C lineage were owned by the same beekeeper who recently imported European queens into Mauritius. Larger data collection using both the ND2 and the COI-COII markers detected the presence of up to four subspecies maternal lineages in the Mascarene archipelago. A diverse assembly of *A. m. unicolor* (A), *A. m. scutellata*-like (A), *A. m. carnica* (C) *A. m. mellifera* (M) haplotypes were found in La Réunion; *A. m. unicolor* (A), *A. m. scutellata/adansonii*-like (A), *A. m. carnica* (C) *A. m. ligustica* (C) in Mauritius; and *A. m. carnica* (C) *A. m. ligustica* (C) in Rodrigues.

All together i) the importation histories, ii) the beekeeping practices (selection of preferable European commercial strains or local colonies) and iii) the availability of native habitat remaining (75% to 0%) may explain the great differences among the three islands. Such differences among geographically close islands have already been described for the Macaronesia archipelagos (Canary Islands, Madeira, Azores) where beekeeping importation is responsible for the high mitochondrial diversity and shift over time [18]. Here, the highest proportion of exotic C and M lineages were found on Mauritius and Rodrigues which were also the only islands importing commercial strains until 2011 [72]. *A. m. ligustica* sequences were detected in Mauritius validating the report of recent introduction of commercial strains from Australia [73, 74]. According to government report, the current honey bee population from Rodrigues was suspected to be strongly related to its sister island population, due to their relations for commercial trade and country politics [40]. Yet, results suggested that importations may come from different commercial stock *of A. m. carnica and A. m. ligustica*. Earliest inference on demographic scenarios in Rodrigues estimated an bottleneck event 49 generations ago, which is more consistent with importation from a different stock as it was reported for queens from the USA in 1980s [44]. In La Réunion, importation has been forbidden since 1982 [75] following important colony damages caused by acarine mites and nosema infections. Unreported and prohibited importation could have happened during this introduction-free time interval, but given that numerous European importations were recorded during the First World War [41], rare frequencies of C and M lineages could rather be residual effects from before that limitation. Surprisingly, no Italian *A. m. ligustica* colonies have been detected in La Réunion despite introduction records [41]. The non-detection of *A. m. ligustica* individuals could be due to sampling bias assuming that colonies are extremely rare and isolated from large scale and frequent migratory movements induced by beekeeping practices across the island. A more likely hypothesis would be that imported temperate honey bees could have been less competitive than African colonies under tropical environmental pressures. In La Réunion, it has been estimated that 75% of original habitats remains while only 2% and 0% have been reported in Mauritius and Rodrigues, respectively [36, 76, 77]. These differences in wild habitats availability and floral resources could also have played a role in the persistence and survival of temperate colonies. Variations in behavior, physiology and gene expression have been demonstrated in honey bees, including among temperate (overwintering) and tropical subspecies [2, 11, 78]. As an example, temperature variations are believed to have played a role in the divergence between *A. m. carnica* and *A. m. macedonica* rather than the Carpathian geographic barrier [69]. In addition to environmental factors, genetic origin could have also influenced the survival of colonies [79].

Cohabitation of divergent lineages and subspecies into the island could have also led to hybridization phenomenon. The phenomenon of Africanization which is well recorded in the New World as the quick replacement of European population by African colonies [21, 80] could also have occurred in the island yet without resulting in any known aggressive behaviour. In order to check the complete absence of some imported colonies, it would be necessary to look through another perspective by using bi-parental markers. Rather than mitochondrial DNA which only informs about maternal history, nuclear markers would allow the detection of the contribution of drones.

### Is the African lineage found in insular SWIO populations identical to continental populations?

The tropical African A_I_ sub-lineage was detected in all continental African populations (except in Egypt) and in 10 of the 11 studied SWIO islands. Both the non-coding COI-COII and the coding ND2 regions analysis demonstrated that African A_I_ haplotypes exclusively retrieved in the SWIO formed a distinct mitochondrial group when compared to continental African populations. These haplotypes were considered as characteristics of *A. m. unicolor* and confirmed once again its genetic differentiation from the other African subspecies [7, 8]. Formerly reported ND2 region divergence level between A_I_ (comprising *A. m. adansonii, A. m. capensis, A. m. monticola* and *A. m. scutellata* samples) and A_III_ sub-lineages (*A. m. intermissa, A.m. iberiensis, A. m. sicula* and *A. m. sahariensis*) in Africa (0.62% ± 0.11) was even lower than the distance found between the insular and continental groups of A_I_ sub-lineage (1.44% ± 0.48) [4]. This divergence level is even comparable to the one observed between the latest confirmed Z sub-lineage [9, 28] and the others A_I_ and A_III_.

Previous estimations based on mitochondrial sequences have estimated the split between A, C, M and O honey bee evolutionary lineages to have occurred either around 1,000,000 years ago using the COI-COII region [3] or 670,000 years ago using the ND2 region [4]. However, the reliability of these estimates remain questionable, as they were calculated using the calibrated rate of *Drosophila* (2% per million years) [81]) since no calibrated clock for honey bees exists. Recent study of genomic variation based on 8.5 million SNPs and focusing on 10 honey bee subspecies has refined the estimation for the evolutionary lineages split to 300,000 to 200,000 years ago [11]. The same study estimates the split among the three major African subspecies *A. m. scutellata*, *A. m. adansonii*, *A. m. capensis* to have occurred 32,000 to 22,000 years ago. Regarding mtDNA divergence levels obtained here for ND2 gene, it is reasonable to think that the split between the A_I_ insular group associated with *A. m. unicolor* and the African continental subspecies complex occurred in a similar period (i.e. during the last glaciation). First human colonization and settlement in the SWIO was estimated to take place at least 5000 years ago in Madagascar (fossil, tip dating on mitochondrial genome) [82–84] and more recently, in the 1770s for the Seychelles archipelago [39]. Therefore, the colonization of Madagascar and the surrounding archipelagos by the SWIO A_I_ honey bees took place well before human settlement and could be considered as indigenous to the area. Additional divergence might be the result of restricted gene flow between continental and insular honey bee populations. Mutation steps (ND2) among SWIO A_I_ and continental African sequences also involved non-synonymous substitutions impacting the protein assembly, and could possibly affect the survival of a colony in the environment.

### Mitochondrial diversity can provide hints on colonization and diversification processes of the SWIO honey bees

All the SWIO islands (except Rodrigues) shared a common and predominant African COI-COII haplotype A1_MAD3, already described in *A. m. unicolor* population from Madagascar [31]. In this study, A1_MAD3 was also described for the first time in Senegal and Malawi and it can be supposed that this particular sequence i) appeared by homoplasy in geographical distant populations from the African continent and Madagascar, or ii) was originally present in African populations that colonized Madagascar. As this sequence was found predominant in Madagascar, its frequency and distribution all over the island is in favor of the hypothesis of a bottleneck event in which the first individuals that colonized the island possessed this sequence and then spread. This hypothesis is further supported by the star-like pattern observed in COI-COII haplotype network in Madagascar, in which 13 other highly similar sequences (99%) radiated from the node A1_MAD3 [31]. This pattern is typical of demographic expansion that could follow a colonization event in islands from a continental population of origin [85]. Such a pattern was reported for the endemic bat *Nyctalus azoreum*, for which ancestors likely colonized the Azores islands from continental European populations in the Pleistocene [86, 87]. Additionally, the star-like network associated with phylogeny of *Cyt b* and 12S rRNA in the study of genetic diversity in *Tarentola* geckos indicated a long oceanic dispersal event from Canary Islands to the Cape Verde archipelago [88]. The star-shaped topology was also observed for all the islands of the Mascarene (except Rodrigues), Seychelles and Comoros archipelagos for which the presence of *A. m. unicolor* is unequivocally established. Each island exhibited one or several private COI-COII sequences that could have originated from A1_MAD3. To detect such a pattern, colonization events need to be followed over a longer time period of gene flow restriction so that each island population can independently evolve and genetically differentiate. These results support the hypothesis that *A. m. unicolor* is indigenous to the SWIO islands and could have colonized most of the nearby archipelagos from Madagascar (Mascarenes, Seychelles). Nevertheless, nucleotide divergence between the COI-COII P_0_Q private sequences is too low to estimate the time of the colonization event of the archipelagos. Implementation of further markers will be necessary to assess the demographic history and confirm the ancient presence of *A. m. unicolor* in the area.

The Comoros archipelago featured a high proportion of African private haplotypes regarding sampling size (*n*_COI-COII_ = 5 and *n*_ND2_ = 3) and also presents individuals with intermediate sequences between continental and insular populations (Mohéli). Considering the young geological history of these islands, the African subspecies divergence time (30,000 to 20,000 years before present [11]), and the variation of ocean levels [89], honey bee could have first colonized Comoros from the East African coast, then colonized Madagascar and others archipelagos. As the sea-levels dramatically lowered, new pathways appeared which would have allowed honey bees to reach the islands following a *stepping-stone*. This model colonization model has been demonstrated for insect colonization of the Mascarene [90] or even for long owls colonization of several islands in the Indian Ocean [91]. In Grande Comore, the closest island to the East coast of Africa one of the private COI-COII haplotype was the only described haplotype as transiting point from Africa to SWIO. This result supports the idea that the Comoros archipelago could form a natural contact interface between continental African subspecies and insular *A. m. unicolor*. In order to confirm this hypothesis, it is necessary to better know the genetic diversity of potential source populations in places such as Malawi. Ultimately, African honey bees from Comoros may originate from Madagascar and mainland Africa populations closely related to *A. m. litorea* subspecies.

## Conclusion

This study sets the first global mitochondrial genetic basis for the honey bee in the South West Indian Ocean islands, and more particularly in the Mascarenes, Seychelles and Comoros archipelagos. Both COI-COII and ND2 markers revealed congruent findings and implementation of potential source populations, confirming 1) the presence of three evolutionary lineages African A and both European C and M groups, 2) the presence and predominance of a private African A_I_ sub-lineage mitochondrial group in the SWIO archipelagos and Madagascar indicating *A. m. unicolor* presence, and 3) an ancient split between continental and insular African haplotypes groups supporting a colonization older than human settlement according to divergence levels. Regarding the mitochondrial diversity and structure, the native area of *A. m. unicolor* is no longer restricted to its first described endemic range in Madagascar [2]. This area is now enlarged and encompasses the nearby archipelagos. Nevertheless, it is necessary to improve our knowledge about these particular populations especially in the Mascarene islands where different lineages were put in contact. Given that honey bee subspecies readily hybridize, it will be interesting to examine potential introgression in the nuclear patrimony, which the mitochondrial markers cannot reflect due their maternal inheritance. Also, the study of the SWIO population’s genomic diversity could help to better understand and infer the demographic history of the honey bees, identifying sources populations and the evolution of the populations in archipelagos more broadly.

## Additional Files

Additional file 1: Figure S1. Map of the 83 sampling sites (purple circles) in La Réunion and distribution of the 10 detected haplotypes of the COI-COII intergenic region. **Figure S2.** Map of the 24 sampling sites (purple circles) in Mauritius and distribution of the 8 detected haplotypes of the COI-COII intergenic region. **Figure S3**. Minimal Spanning Tree based on 16 European lineages haplotypes of the honey bee COI-COII intergenic region. **Table S2** Distribution and occurrence of the 34 haplotypes of the partial ND2 gene for each SWIO island. **Table S3** Result of AMOVA (Analysis of molecular variance) between population groups based on location and COI-COII markers. A) African colonies from the South West Indian Ocean and African continental populations (*n* > 5) and B) European C colonies from Mascarene archipelago and European continental populations. **Table S4a** Distribution and occurrence of the 34 haplotypes of the partial ND2 gene for each SWIO island. **Table S5** Average of COI-COII sequence divergence percentage (± SD) including insertion/deletions within (in bold) and between mitochondrial groups of honey bees defined according to haplotype network. (DOCX 8618 kb)

Additional file 2: Table S1. Sampling details including location, geo-coordinates, COI-COII and ND2 sequences information for each individual (XLSX 104 kb)

## Abbreviations

COI: Cytochrome oxidase subunit I gene
COII: Cytochrome oxidase subunit II gene
mtDNA: Mitochondrial DNA
Myr: Millions of years
ND2: NADH-dehydrogenase subunit 2 gene
PCR-RFLP: Polymerase Chain Reaction - Restriction Fragment Length Polymorphism
SWIO: South West Indian Ocean
