# Supplementary material for: Large-scale mitochondrial DNA analysis of native honey bee Apis mellifera populations reveals a new African subgroup private to the South West Indian Ocean islands
Source: BMC Genet. 2017 Jun 2;18:53. doi: 10.1186/s12863-017-0520-8 (PMC5457595; doi:10.1186/s12863-017-0520-8)
Supplement: Supplementary file 1 — Map of the 83 sampling sites (purple circles) in La Réunion and distribution of the 10 detected haplotypes of the COI-COII intergenic region. Figure S2. Map of the 24 sampling sites (purple circles) in Mauritius and distribution of the 8 detected haplotypes of the COI-COII intergenic region. Figure S3. Minimal Spanning Tree based on 16 European lineages haplotypes of the honey bee COI-COII intergenic region. Table S2 Distribution and occurrence of the 34 haplotypes of the partial ND2 gene for each SWIO island. Table S3 Result of AMOVA (Analysis of molecular variance) between population groups based on location and COI-COII markers. A) African colonies from the South West Indian Ocean and African continental populations (n > 5) and B) European C colonies from Mascarene archipelago and European continental populations. Table S4a Distribution and occurrence of the 34 haplotypes of the partial ND2 gene for each SWIO island. Table S5 Average of COI-COII sequence divergence percentage (± SD) including insertion/deletions within (in bold) and between mitochondrial groups of honey bees defined according to haplotype network. (DOCX 8618 kb) [file 12863_2017_520_MOESM1_ESM.docx]

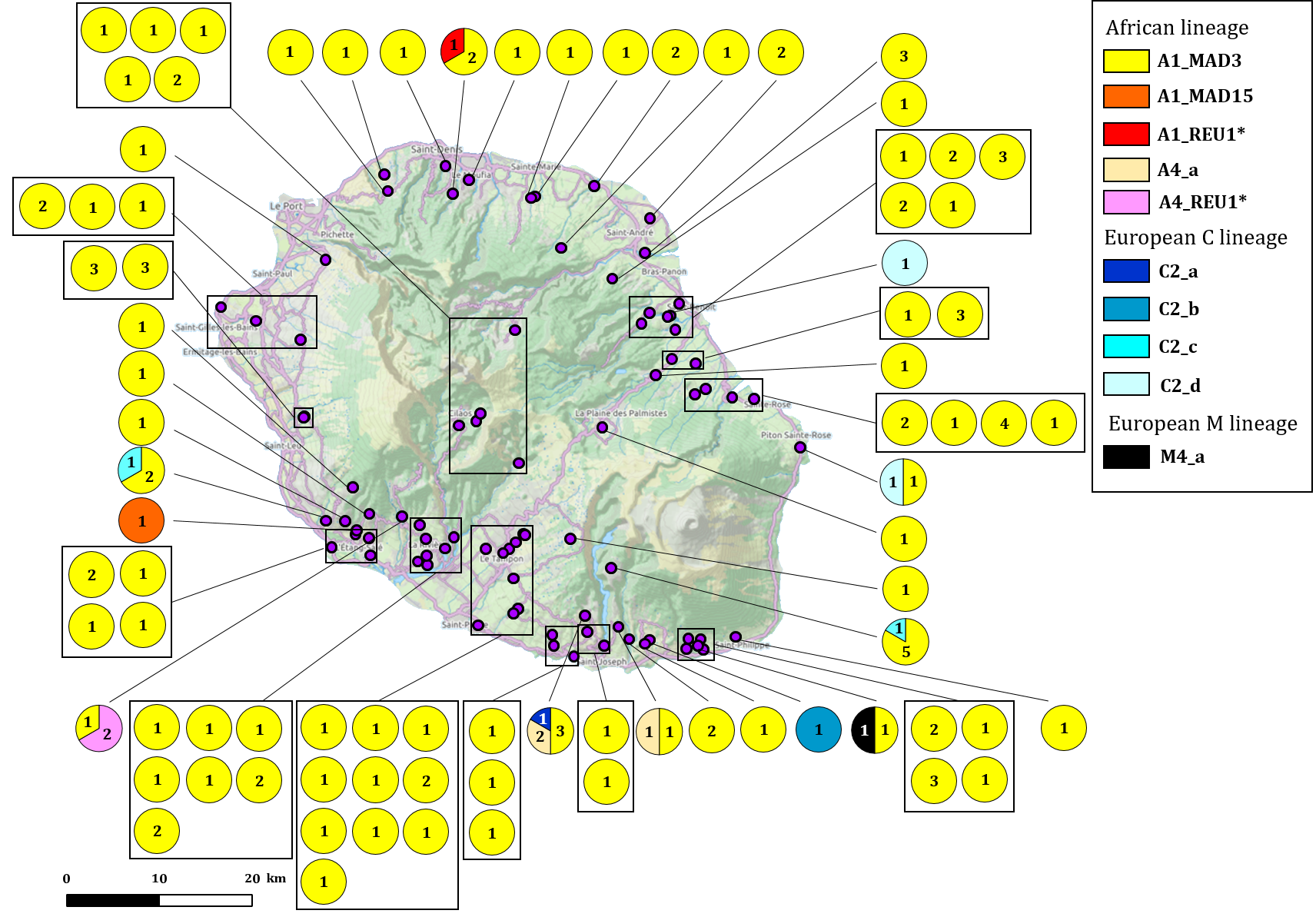


**Figure S1 Map of the 83 sampling sites (purple circles) in La Réunion and distribution of the 10 detected haplotypes of the COI-COII intergenic region.** Each diagram represents the frequencies of the haplotype detected and the number of individuals per haplotype is indicated. Map was constructed using QGIS and Open Landscape Layer from www.opencyclemap.org.

**
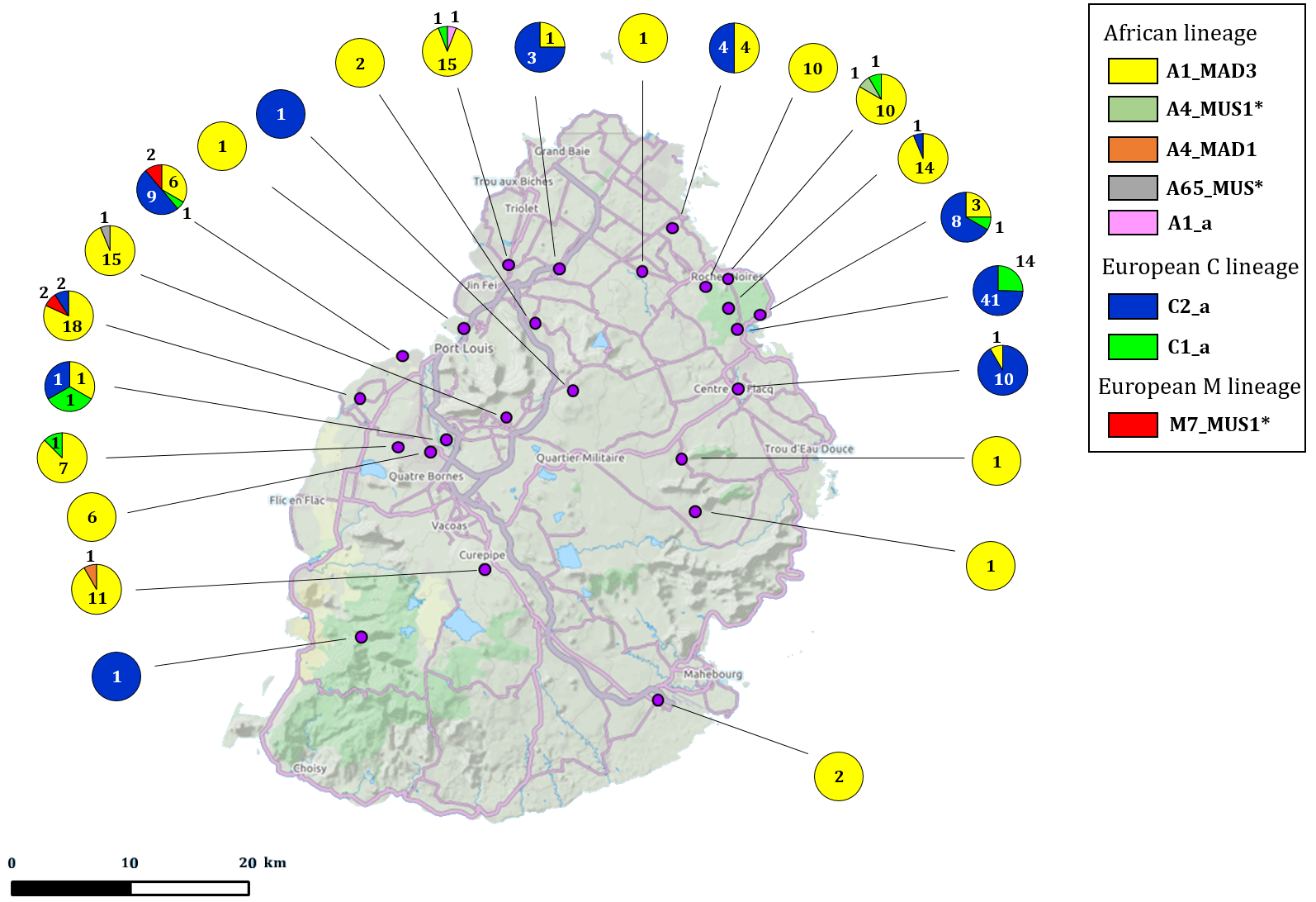
**

**Figure S2 Map of the 24 sampling sites (purple circles) in Mauritius and distribution of the 8 detected haplotypes of the COI-COII intergenic region.** Each diagram represents the frequencies of the haplotype detected and the number of individuals per haplotype is indicated. Map was constructed using QGIS and Open Landscape Layer from www.opencyclemap.org.

**
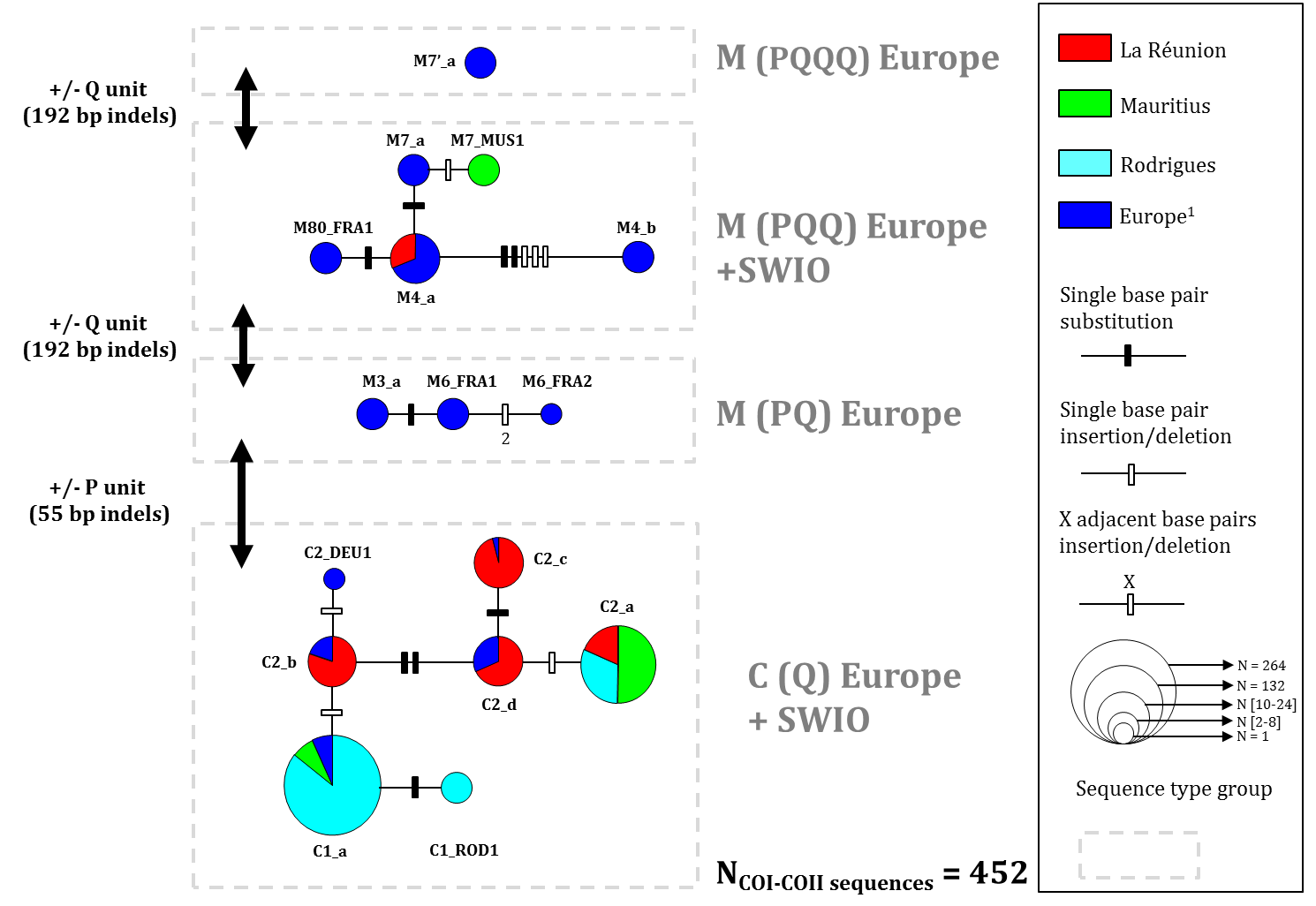
**

**Figure S3 Minimal Spanning Tree based on 16 European lineages haplotypes of the honeybee COI-COII intergenic region.** The haplotype network was based on 15 different haplotypes retrieved from sequencing 452 COI-COII of mtDNA from honeybees of the South West Indian Ocean (SWIO) islands, Africa, Europe. Each circle represents a haplotype, its size indicates frequencies and each color indicates population origin. Substitution and insertion/deletion steps between haplotypes are shown by hatch mark.

Europe^1^: Portugal, Spain, France, Germany, Switzerland, Italy and Greece

**Table S2 Distribution and occurrence of the 84 described COI-COII intergenic region haplotypes for each sampled population**. Population details are given per islands in the South West Indian Ocean, and per country in Africa and Europe. Sequences are ordered by evolutionary lineages A: P_0_Q, P_0_QQ, P_1_QQ and P_1_QQQ; M lineage: PQ, PQQ and PQQQ and C lineage: Q.

|  |  | La Réunion | Mauritius | Rodrigues | Mahé | Praslin | La Digue | Grande Comore | Anjouan | Mohéli | Mayotte | Madagascar | Egypt | Senegal | Chad | CAF | São Tomé | Gabon | Uganda | Tanzania | Malawi | Zimbabwe | Mozambique | South Africa | Portugal | Spain | France | Germany | Switzerland | Italy | Greece |
| --- | --- | --- | --- | --- | --- | --- | --- | --- | --- | --- | --- | --- | --- | --- | --- | --- | --- | --- | --- | --- | --- | --- | --- | --- | --- | --- | --- | --- | --- | --- | --- |
| P_0_Q | A1_a |  | 1 |  |  |  |  |  |  |  |  |  |  |  |  | 1 |  |  |  | 7 | 2 |  | 1 | 2 |  |  |  |  |  |  |  |
|  | A1_b |  |  |  |  |  |  |  |  |  |  |  |  |  |  | 1 |  |  |  | 1 |  |  |  |  | 2 |  |  |  |  |  |  |
|  | A1_MAD3 | 116 | 130 |  | 46 | 43 | 22 | 15 | 21 | 10 | 19 | 143 |  | 2 |  |  |  |  |  |  | 2 |  |  |  |  |  |  |  |  |  |  |
|  | A1_MAD4 |  |  |  |  |  |  |  |  |  |  | 1 |  |  |  |  |  |  |  |  |  |  |  |  |  |  |  |  |  |  |  |
|  | A1_MAD5 |  |  |  |  |  |  |  |  |  |  | 2 |  |  |  |  |  |  |  |  |  |  |  |  |  |  |  |  |  |  |  |
|  | A1_MAD6 |  |  |  |  |  |  |  |  |  |  | 1 |  |  |  |  |  |  |  |  |  |  |  |  |  |  |  |  |  |  |  |
|  | A1_MAD7 |  |  |  |  |  |  |  |  |  |  | 1 |  |  |  |  |  |  |  |  |  |  |  |  |  |  |  |  |  |  |  |
|  | A1_MAD8 |  |  |  |  |  |  |  |  |  |  | 1 |  |  |  |  |  |  |  |  |  |  |  |  |  |  |  |  |  |  |  |
|  | A1_MAD9 |  |  |  |  |  |  |  |  |  |  | 2 |  |  |  |  |  |  |  |  |  |  |  |  |  |  |  |  |  |  |  |
|  | A1_MAD10 |  |  |  |  |  |  |  |  |  |  | 3 |  |  |  |  |  |  |  |  |  |  |  |  |  |  |  |  |  |  |  |
|  | A1_MAD11 |  |  |  |  |  |  |  |  |  |  | 1 |  |  |  |  |  |  |  |  |  |  |  |  |  |  |  |  |  |  |  |
|  | A1_MAD12 |  |  |  |  |  |  |  |  |  | 1 | 1 |  |  |  |  |  |  |  |  |  |  |  |  |  |  |  |  |  |  |  |
|  | A64 (A1_MAD13) |  |  |  |  |  |  |  |  |  |  | 2 |  |  |  |  |  |  |  |  |  |  |  |  |  |  |  |  |  |  |  |
|  | A1_MAD14 |  |  |  |  |  |  |  |  |  |  | 1 |  |  |  |  |  |  |  |  |  |  |  |  |  |  |  |  |  |  |  |
|  | A1_MAD15 | 1 |  |  |  |  |  | 7 | 1 |  |  | 2 |  |  |  |  |  |  |  |  |  |  |  |  |  |  |  |  |  |  |  |
|  | A1_MAD16 |  |  |  |  |  |  |  |  |  |  | 1 |  |  |  |  |  |  |  |  |  |  |  |  |  |  |  |  |  |  |  |
|  | A1_REU1 | 1 |  |  |  |  |  |  |  |  |  |  |  |  |  |  |  |  |  |  |  |  |  |  |  |  |  |  |  |  |  |
|  | A1_SEY1 |  |  |  | 3 |  |  |  |  |  |  |  |  |  |  |  |  |  |  |  |  |  |  |  |  |  |  |  |  |  |  |
|  | A1_SEY2 |  |  |  | 1 |  |  |  |  |  |  |  |  |  |  |  |  |  |  |  |  |  |  |  |  |  |  |  |  |  |  |
|  | A1_GCO1 |  |  |  |  |  |  | 3 |  |  |  |  |  |  |  |  |  |  |  |  |  |  |  |  |  |  |  |  |  |  |  |
|  | A1_GCO2 |  |  |  |  |  |  | 4 |  |  |  |  |  |  |  |  |  |  |  |  |  |  |  |  |  |  |  |  |  |  |  |
|  | A1_ANJ1 |  |  |  |  |  |  |  | 5 |  |  |  |  |  |  |  |  |  |  |  |  |  |  |  |  |  |  |  |  |  |  |
|  | A1_MYT1 |  |  |  |  |  |  |  |  |  | 2 |  |  |  |  |  |  |  |  |  |  |  |  |  |  |  |  |  |  |  |  |
|  | A1_MYT2 |  |  |  |  |  |  |  |  |  | 1 |  |  |  |  |  |  |  |  |  |  |  |  |  |  |  |  |  |  |  |  |
|  | A1_MYT3 |  |  |  |  |  |  |  |  |  | 1 |  |  |  |  |  |  |  |  |  |  |  |  |  |  |  |  |  |  |  |  |
|  | A1_CAF1 |  |  |  |  |  |  |  |  |  |  |  |  |  |  | 3 |  |  |  |  |  |  |  |  |  |  |  |  |  |  |  |
|  | A1_CAF2 |  |  |  |  |  |  |  |  |  |  |  |  |  |  | 1 |  |  |  |  |  |  |  |  |  |  |  |  |  |  |  |
|  | A1_GAB1 |  |  |  |  |  |  |  |  |  |  |  |  |  |  |  |  | 2 |  |  |  |  |  |  |  |  |  |  |  |  |  |
|  | A1_TZA1 |  |  |  |  |  |  |  |  |  |  |  |  |  |  |  |  |  |  | 1 |  |  |  |  |  |  |  |  |  |  |  |
|  | A1_MOZ1 |  |  |  |  |  |  |  |  |  |  |  |  |  |  |  |  |  |  |  |  |  | 1 |  |  |  |  |  |  |  |  |
|  | A65_MUS1 |  | 1 |  |  |  |  |  |  |  |  |  |  |  |  |  |  |  |  |  |  |  |  |  |  |  |  |  |  |  |  |
|  | Z7_EGY1 |  |  |  |  |  |  |  |  |  |  |  | 1 |  |  |  |  |  |  |  |  |  |  |  |  |  |  |  |  |  |  |
|  | **TOTAL** | **118** | **132** |  | **50** | **43** | **22** | **29** | **27** | **10** | **24** | **162** | **1** | **2** |  | **6** |  | **2** |  | **9** | **4** |  | **2** | **2** | **2** |  |  |  |  |  |  |

**Table S2**(following, part2/3)

|  |  | La Réunion | Mauritius | Rodrigues | Mahé | Praslin | La Digue | Grande Comore | Anjouan | Mohéli | Mayotte | Madagascar | Egypt | Senegal | Chad | CAF | São Tomé | Gabon | Uganda | Tanzania | Malawi | Zimbabwe | Mozambique | South Africa | Portugal | Spain | France | Germany | Switzerland | Italy | Greece |
| --- | --- | --- | --- | --- | --- | --- | --- | --- | --- | --- | --- | --- | --- | --- | --- | --- | --- | --- | --- | --- | --- | --- | --- | --- | --- | --- | --- | --- | --- | --- | --- |
| P_0_QQ | A4_a | 3 |  |  |  |  |  |  |  |  |  |  |  |  |  | 1 | 7 |  |  | 1 |  |  |  |  |  |  |  |  |  |  |  |
|  | A4_b |  |  |  |  |  |  |  |  |  |  |  |  |  |  |  |  |  |  |  |  | 5 |  | 8 |  |  |  |  |  |  |  |
|  | A4_c |  |  |  |  |  |  |  |  |  |  |  |  |  | 1 | 1 |  |  |  |  |  | 4 |  |  |  |  |  |  |  |  |  |
|  | A4_MAD1 |  | 1 |  |  |  |  |  |  |  |  | 1 |  |  |  |  |  |  |  |  |  |  |  |  |  |  |  |  |  |  |  |
|  | A4_MAD2 |  |  |  |  |  |  |  |  |  |  | 1 |  |  |  |  |  |  |  |  |  |  |  |  |  |  |  |  |  |  |  |
|  | A4_REU1 | 2 |  |  |  |  |  |  |  |  |  |  |  |  |  |  |  |  |  |  |  |  |  |  |  |  |  |  |  |  |  |
|  | A4_MUS1 |  | 1 |  |  |  |  |  |  |  |  |  |  |  |  |  |  |  |  |  |  |  |  |  |  |  |  |  |  |  |  |
|  | A4_SEN1 |  |  |  |  |  |  |  |  |  |  |  |  | 1 |  |  |  |  |  |  |  |  |  |  |  |  |  |  |  |  |  |
|  | A4_SEN2 |  |  |  |  |  |  |  |  |  |  |  |  | 1 |  |  |  |  |  |  |  |  |  |  |  |  |  |  |  |  |  |
|  | A4_STP1 |  |  |  |  |  |  |  |  |  |  |  |  |  |  |  | 1 |  |  |  |  |  |  |  |  |  |  |  |  |  |  |
|  | A4_CAF1 |  |  |  |  |  |  |  |  |  |  |  |  |  |  | 2 |  |  |  |  |  |  |  |  |  |  |  |  |  |  |  |
|  | A4_CAF2 |  |  |  |  |  |  |  |  |  |  |  |  |  |  | 1 |  |  |  |  |  |  |  |  |  |  |  |  |  |  |  |
|  | A4_GAB1 |  |  |  |  |  |  |  |  |  |  |  |  |  |  |  |  | 1 |  |  |  |  |  |  |  |  |  |  |  |  |  |
|  | A4_TCD1 |  |  |  |  |  |  |  |  |  |  |  |  |  | 1 |  |  |  |  |  |  |  |  |  |  |  |  |  |  |  |  |
|  | A4_ZIM1 |  |  |  |  |  |  |  |  |  |  |  |  |  |  |  |  |  |  |  |  | 1 |  |  |  |  |  |  |  |  |  |
|  | A4_ZIM2 |  |  |  |  |  |  |  |  |  |  |  |  |  |  |  |  |  |  |  |  | 3 |  |  |  |  |  |  |  |  |  |
|  | A4_ZIM3 |  |  |  |  |  |  |  |  |  |  |  |  |  |  |  |  |  |  |  |  | 1 |  |  |  |  |  |  |  |  |  |
|  | A4_ZAF1 |  |  |  |  |  |  |  |  |  |  |  |  |  |  |  |  |  |  |  |  |  |  | 3 |  |  |  |  |  |  |  |
|  | A4_ZAF2 |  |  |  |  |  |  |  |  |  |  |  |  |  |  |  |  |  |  |  |  |  |  | 1 |  |  |  |  |  |  |  |
|  | A4_ZAF3 |  |  |  |  |  |  |  |  |  |  |  |  |  |  |  |  |  |  |  |  |  |  | 1 |  |  |  |  |  |  |  |
|  | A4_ZAF4 |  |  |  |  |  |  |  |  |  |  |  |  |  |  |  |  |  |  |  |  |  |  | 1 |  |  |  |  |  |  |  |
|  | A4_ZAF5 |  |  |  |  |  |  |  |  |  |  |  |  |  |  |  |  |  |  |  |  |  |  | 1 |  |  |  |  |  |  |  |
|  | A4_ZAF6 |  |  |  |  |  |  |  |  |  |  |  |  |  |  |  |  |  |  |  |  |  |  | 1 |  |  |  |  |  |  |  |
|  | A4_ZAF7 |  |  |  |  |  |  |  |  |  |  |  |  |  |  |  |  |  |  |  |  |  |  | 1 |  |  |  |  |  |  |  |
|  | A4_ZAF8 |  |  |  |  |  |  |  |  |  |  |  |  |  |  |  |  |  |  |  |  |  |  | 1 |  |  |  |  |  |  |  |
|  | A4_ZAF9 |  |  |  |  |  |  |  |  |  |  |  |  |  |  |  |  |  |  |  |  |  |  | 1 |  |  |  |  |  |  |  |
|  | A6_TCD1 |  |  |  |  |  |  |  |  |  |  |  |  |  | 1 |  |  |  |  |  |  |  |  |  |  |  |  |  |  |  |  |
|  | A66_STP1 |  |  |  |  |  |  |  |  |  |  |  |  |  |  |  | 1 |  |  |  |  |  |  |  |  |  |  |  |  |  |  |
|  | A66_ZAF1 |  |  |  |  |  |  |  |  |  |  |  |  |  |  |  |  |  |  |  |  |  |  | 1 |  |  |  |  |  |  |  |
|  | A67_UGA1 |  |  |  |  |  |  |  |  |  |  |  |  |  |  |  |  |  | 1 |  |  |  |  |  |  |  |  |  |  |  |  |
|  | Z2_SEY1 |  |  |  |  | 4 | 4 |  |  |  |  |  |  |  |  |  |  |  |  |  |  |  |  |  |  |  |  |  |  |  |  |
|  | **TOTAL** | **5** | **2** |  |  | **4** | **4** |  |  |  |  | **2** |  | **2** | **3** | **5** | **9** | **1** | **1** | **1** |  | **14** |  | **20** |  |  |  |  |  |  |  |

**Table S2**(following, part3/3)

|  |  | La Réunion | Mauritius | Rodrigues | Mahé | Praslin | La Digue | Grande Comore | Anjouan | Mohéli | Mayotte | Madagascar | Egypt | Senegal | Chad | CAF | São Tomé | Gabon | Uganda | Tanzania | Malawi | Zimbabwe | Mozambique | South Africa | Portugal | Spain | France | Germany | Switzerland | Italy | Greece |
| --- | --- | --- | --- | --- | --- | --- | --- | --- | --- | --- | --- | --- | --- | --- | --- | --- | --- | --- | --- | --- | --- | --- | --- | --- | --- | --- | --- | --- | --- | --- | --- |
| P_1_QQ | A11_PRT1 |  |  |  |  |  |  |  |  |  |  |  |  |  |  |  |  |  |  |  |  |  |  |  | 5 | 2 |  |  |  |  |  |
|  | A11_PRT2 |  |  |  |  |  |  |  |  |  |  |  |  |  |  |  |  |  |  |  |  |  |  |  | 1 |  |  |  |  |  |  |
|  | A14_PRT1 |  |  |  |  |  |  |  |  |  |  |  |  |  |  |  |  |  |  |  |  |  |  |  | 1 |  |  |  |  |  |  |
| P_1_QQ | A16_PRT1 |  |  |  |  |  |  |  |  |  |  |  |  |  |  |  |  |  |  |  |  |  |  |  | 1 |  |  |  |  |  |  |
|  | A16_PRT2 |  |  |  |  |  |  |  |  |  |  |  |  |  |  |  |  |  |  |  |  |  |  |  | 2 |  |  |  |  |  |  |
|  | **TOTAL** |  |  |  |  |  |  |  |  |  |  |  |  |  |  |  |  |  |  |  |  |  |  |  | **10** | **2** |  |  |  |  |  |
|  |  |  |  |  |  |  |  |  |  |  |  |  |  |  |  |  |  |  |  |  |  |  |  |  |  |  |  |  |  |  |  |
| PQ | M3_a |  |  |  |  |  |  |  |  |  |  |  |  |  |  |  |  |  |  |  |  |  |  |  |  |  |  |  |  | 2 |  |
|  | M6_FRA1 |  |  |  |  |  |  |  |  |  |  |  |  |  |  |  |  |  |  |  |  |  |  |  |  |  | 2 |  |  |  |  |
|  | M6_FRA2 |  |  |  |  |  |  |  |  |  |  |  |  |  |  |  |  |  |  |  |  |  |  |  |  |  | 1 |  |  |  |  |
| PQQ | M4_a | 1 |  |  |  |  |  |  |  |  |  |  |  |  |  |  |  |  |  |  |  |  |  |  |  |  | 11 |  |  |  |  |
|  | M4_b |  |  |  |  |  |  |  |  |  |  |  |  |  |  |  |  |  |  |  |  |  |  |  |  |  | 2 |  |  |  |  |
|  | M80_FRA1 |  |  |  |  |  |  |  |  |  |  |  |  |  |  |  |  |  |  |  |  |  |  |  |  |  | 3 |  |  |  |  |
|  | M7_a |  |  |  |  |  |  |  |  |  |  |  |  |  |  |  |  |  |  |  |  |  |  |  |  |  |  |  |  | 7 |  |
|  | M7_MUS1 |  | 4 |  |  |  |  |  |  |  |  |  |  |  |  |  |  |  |  |  |  |  |  |  |  |  |  |  |  |  |  |
| PQQQ | M7'_a |  |  |  |  |  |  |  |  |  |  |  |  |  |  |  |  |  |  |  |  |  |  |  |  | 1 | 4 |  |  | 1 |  |
|  | **TOTAL** | **1** | **4** |  |  |  |  |  |  |  |  |  |  |  |  |  |  |  |  |  |  |  |  |  |  | **1** | **23** |  |  | **10** |  |
|  |  |  |  |  |  |  |  |  |  |  |  |  |  |  |  |  |  |  |  |  |  |  |  |  |  |  |  |  |  |  |  |
| Q | C1_a |  | 20 | 226 |  |  |  |  |  |  |  |  |  |  |  |  |  |  |  |  |  |  |  |  |  |  | 4 |  | 2 | 12 |  |
|  | C1_ROD1 |  |  | 2 |  |  |  |  |  |  |  |  |  |  |  |  |  |  |  |  |  |  |  |  |  |  |  |  |  |  |  |
|  | C2_a | 1 | 81 | 50 |  |  |  |  |  |  |  |  |  |  |  |  |  |  |  |  |  |  |  |  |  |  |  |  |  |  |  |
|  | C2_b | 1 |  |  |  |  |  |  |  |  |  |  |  |  |  |  |  |  |  |  |  |  |  |  |  |  | 1 | 1 |  |  |  |
|  | C2_c | 2 |  |  |  |  |  |  |  |  |  |  |  |  |  |  |  |  |  |  |  |  |  |  |  |  |  | 1 |  |  |  |
|  | C2_d | 2 |  |  |  |  |  |  |  |  |  |  |  |  |  |  |  |  |  |  |  |  |  |  |  |  |  |  |  |  | 6 |
|  | C2_DEU1 |  |  |  |  |  |  |  |  |  |  |  |  |  |  |  |  |  |  |  |  |  |  |  |  |  |  | 1 |  |  |  |
|  | **TOTAL** | **6** | **101** | **278** |  |  |  |  |  |  |  |  |  |  |  |  |  |  |  |  |  |  |  |  |  |  | **5** | **3** | **2** | **12** | **6** |

**Table S3 Result of AMOVA (Analysis of molecular variance) between population groups based on location and COI-COII markers. A) African colonies from the South West Indian Ocean and African continental populations (*n* > 5) and B) European C colonies from Mascarene archipelago and European continental populations.** φPT values were computed by pairwise genetic distances in GenAlEx version 6.5, and probabilities based on 999 permutations are shown above the diagonal (grey background).

**A)**

| **La Réunion** | **Mauritius** | **Mahé** | **Praslin** | **La Digue** | **Grande Comore** | **Anjouan** | **Mohéli** | **Mayotte** | **Madagascar** | **Central African Rep.** | **Tanzania** |  |  |
| --- | --- | --- | --- | --- | --- | --- | --- | --- | --- | --- | --- | --- | --- |
|  | 0.454 | 0.023 | 0.451 | 0.294 | 0.001 | 0.001 | 0.138 | 0.003 | 0.472 | 0.001 | 0.001 | **La Réunion** | (*n =* 118) |
| 0.000 |  | 0.055 | 0.229 | 0.135 | 0.001 | 0.001 | 0.125 | 0.039 | 0.104 | 0.001 | 0.001 | **Mauritius** | (*n =* 132) |
| 0.036 | 0.020 |  | 0.244 | 0.528 | 0.001 | 0.001 | 0.226 | 0.033 | 0.117 | 0.001 | 0.001 | **Mahé** | (*n =* 50) |
| 0.000 | 0.000 | 0.026 |  | 0.001 | 0.001 | 0.005 | 0.001 | 0.003 | 0.446 | 0.001 | 0.001 | **Praslin** | (*n =* 43) |
| 0.000 | 0.000 | 0.004 | 0.000 |  | 0.003 | 0.061 | 0.001 | 0.487 | 0.381 | 0.001 | 0.001 | **La Digue** | (*n =* 22) |
| 0.286 | 0.260 | 0.170 | 0.194 | 0.129 |  | 0.001 | 0.103 | 0.003 | 0.001 | 0.001 | 0.001 | **Grande Comore** | (*n =* 29) |
| 0.280 | 0.243 | 0.157 | 0.188 | 0.122 | 0.129 |  | 0.295 | 0.020 | 0.001 | 0.001 | 0.001 | **Anjouan** | (*n =* 27) |
| 0.000 | 0.000 | 0.000 | 0.000 | 0.000 | 0.070 | 0.062 |  | 0.400 | 0.383 | 0.002 | 0.001 | **Mohéli** | (*n =* 10) |
| 0.077 | 0.059 | 0.036 | 0.045 | 0.010 | 0.102 | 0.091 | 0.000 |  | 0.123 | 0.001 | 0.001 | **Mayotte** | (*n =* 24) |
| 0.000 | 0.002 | 0.006 | 0.000 | 0.000 | 0.157 | 0.140 | 0.000 | 0.017 |  | 0.001 | 0.001 | **Madagascar** | (*n =* 162) |
| 0.967 | 0.960 | 0.922 | 0.937 | 0.892 | 0.811 | 0.828 | 0.813 | 0.842 | 0.928 |  | 0.040 | **Central African Rep.** | (*n =* 6) |
| 0.985 | 0.976 | 0.962 | 0.983 | 0.971 | 0.874 | 0.892 | 0.951 | 0.914 | 0.942 | 0.196 |  | **Tanzania** | (*n =* 9) |

**B)**

| **La Réunion** | **Mauritius** | **Rodrigues** | **France** | **Greece** | **Italy** |  |  |
| --- | --- | --- | --- | --- | --- | --- | --- |
|  | 0.017 | 0.001 | 0.008 | 0.090 | 0.002 | **La Réunion** | (*n =* 6) |
| 0.254 |  | 0.001 | 0.005 | 0.001 | 0.001 | **Mauritius** | (*n =* 101) |
| 0.560 | 0.558 |  | 0.591 | 0.012 | 0.359 | **Rodrigues** | (*n =* 278) |
| 0.658 | 0.634 | 0.000 |  | 0.098 | 0.458 | **France** | (*n =* 5) |
| 0.204 | 0.394 | 0.191 | 0.237 |  | 0.106 | **Greece** | (*n =* 12) |
| 0.758 | 0.669 | 0.024 | 0.040 | 0.351 |  | **Italy** | (*n =* 6) |

**Table S4a Distribution and occurrence of the 34 haplotypes of the partial ND2 gene for each SWIO island.** Only one representant of each combination of ND2 haplotype/population/COI-COII haplotype was represented in the Approximate-Maximum-likelihood tree (Figure 5). Among the 171 individuals sequenced for the partial ND2 gene, 83 different combinations were detected. Number of individuals harboring the same combination is indicated between brackets at the right of COI-COII sequence type. *Newly described sequences.

| ND2 ST | La Réunion | Mauritius | Rodrigues | Mahé | Praslin | La Digue | Grande Comore | Anjouan | Mohéli | Mayotte | Madagascar |
| --- | --- | --- | --- | --- | --- | --- | --- | --- | --- | --- | --- |
| SWIO01* | A1_MAD3 (4) | A1_MAD3 (3) |  | A1_MAD3 (2) | A1_MAD3 (2) | A1_MAD3 (2) | A1_MAD3 (2) | A1_MAD3 (1) |  | A1_MAD3 (1) | A1_MAD3 (8) |
|  | A1_MAD15 (1) | A4_MAD1 (1) |  | A1_SEY1 (2) |  |  | A1_GCO1 (2) | A1_MAD15 (1) |  | A1_MYT1 (1) | A1_MAD5 (2) |
|  | A1_REU1 (1) | A4_MUS1 (1) |  | A1_SEY2 (1) |  |  | A1_GCO2 (2) | A1_ANJ1 (2) |  | A1_MYT2 (1) | A1_MAD6 (1) |
|  | A4_REU1 (1) | A65_MUS1 (1) |  |  |  |  |  |  |  |  | A1_MAD8 (1) |
|  |  |  |  |  |  |  |  |  |  |  | A1_MAD9 (2) |
|  |  |  |  |  |  |  |  |  |  |  | A1_MAD10 (2) |
|  |  |  |  |  |  |  |  |  |  |  | A1_MAD12 (1) |
|  |  |  |  |  |  |  |  |  |  |  | A64 (1) |
|  |  |  |  |  |  |  |  |  |  |  | A1_MAD14 (1) |
|  |  |  |  |  |  |  |  |  |  |  | A1_MAD15 (2) |
|  |  |  |  |  |  |  |  |  |  |  | A1_MAD16 (1) |
|  |  |  |  |  |  |  |  |  |  |  | A4_MAD1 (1) |
| SWIO02* |  |  |  |  |  |  |  |  |  |  | A4_MAD2 (1) |
| SWIO03* |  |  |  |  |  |  | A1_MAD15 (2) |  |  |  |  |
| SWIO04* |  |  |  |  |  |  |  |  |  | A1_MYT3 (1) |  |
| SWIO05* |  |  |  |  |  |  |  | A1_MAD3 (1) |  |  |  |
| AFR01 |  | A1_a (1) |  |  |  |  |  |  |  |  |  |
| AFR02* |  |  |  |  |  |  |  |  |  |  |  |
| AFR03* |  |  |  |  |  |  |  |  |  |  |  |
| AFR04* |  |  |  |  |  |  |  |  |  |  |  |
| AFR05 |  |  |  |  |  |  |  |  |  |  |  |
| AFR06 | A4_a (2) |  |  |  |  |  |  |  |  |  |  |
| AFR07 |  |  |  |  |  |  |  |  |  |  |  |
| AFR08* |  |  |  |  |  |  |  |  |  |  |  |
| AFR09 |  |  |  |  |  |  |  |  |  |  |  |
| AFR10* |  |  |  |  |  |  |  |  |  |  |  |
| AFR11 |  |  |  |  |  |  |  |  |  |  |  |
| AFR12* |  |  |  |  | Z2_SEY1 (2) | Z2_SEY1 (2) |  |  |  |  |  |
| AFR13* |  |  |  |  |  |  |  |  | A1_MAD3 (3) |  |  |
| EUR01* | M4_a (1) |  |  |  |  |  |  |  |  |  |  |
| EUR02* |  |  |  |  |  |  |  |  |  |  |  |
| EUR03 |  |  |  |  |  |  |  |  |  |  |  |
| EUR04* |  |  |  |  |  |  |  |  |  |  |  |
| EUR05* |  | M7_MUS1 (2) |  |  |  |  |  |  |  |  |  |
| EUR06* |  |  |  |  |  |  |  |  |  |  |  |
| EUR07* |  |  | C1_a (2) |  |  |  |  |  |  |  |  |
|  |  |  | C1_Rod1 (2) |  |  |  |  |  |  |  |  |
| EUR08* |  |  |  |  |  |  |  |  |  |  |  |
| EUR09 |  | C1_a (3) |  |  |  |  |  |  |  |  |  |
| EUR10* | C2_a (1) | C2_a (2) | C2_a (1) |  |  |  |  |  |  |  |  |
| EUR11* |  |  | C2_a (1) |  |  |  |  |  |  |  |  |
| EUR12* |  |  |  |  |  |  |  |  |  |  |  |
| EUR13* | C2_b (1) |  |  |  |  |  |  |  |  |  |  |
| EUR14 | C2_c (2) |  |  |  |  |  |  |  |  |  |  |
| EUR15 | C2_d (2) |  |  |  |  |  |  |  |  |  |  |
| EUR16* |  |  |  |  |  |  |  |  |  |  |  |

**Table S4b Distribution and occurrence of the 34 haplotypes of the partial ND2 gene for each African country.**

| ND2 ST | Egypt | Senegal | Chad | CAF | São Tomé | Gabon | Uganda | Tanzania | Malawi | Zimbabwe | Mozambique | South Africa |
| --- | --- | --- | --- | --- | --- | --- | --- | --- | --- | --- | --- | --- |
| SWIO01* |  |  |  |  |  |  |  |  |  |  |  |  |
| SWIO02* |  |  |  |  |  |  |  |  |  |  |  |  |
| SWIO03* |  |  |  |  |  |  |  |  |  |  |  |  |
| SWIO04* |  |  |  |  |  |  |  |  |  |  |  |  |
| SWIO05* |  |  |  |  |  |  |  |  |  |  |  |  |
| AFR01 |  |  | A6_TCD1 (1) | A1_a (1) |  | A4_GAB1 (1) | A67_UGA1 (1) | A1_a (1) | A1_a (1) | A4_b (1) | A1_MOZ1 (1) | A1_a (1) |
|  |  |  |  |  |  |  |  | A1_b (1) |  | A4_c (3) |  | A4_b (2) |
|  |  |  |  |  |  |  |  | A1_TZA1 (1) |  | A4_ZIM1 (1) |  | A4_ZAF8 (1) |
|  |  |  |  |  |  |  |  |  |  | A4_ZIM2 (2) |  |  |
|  |  |  |  |  |  |  |  |  |  | A4_ZIM3 (1) |  |  |
| AFR02* |  |  |  | A1_CAF1 (2) |  |  |  |  |  |  |  |  |
| AFR03* |  |  |  | A4_CAF1 (1) |  |  |  |  |  |  |  |  |
| AFR04* |  |  |  |  |  |  |  |  |  |  | A1_a (1) |  |
| AFR05 |  |  |  |  |  |  |  |  |  |  |  | A4_ZAF1 (1) |
|  |  |  |  |  |  |  |  |  |  |  |  | A4_ZAF2 (1) |
|  |  |  |  |  |  |  |  |  |  |  |  | A4_ZAF3 (1) |
| AFR06 |  |  |  | A4_a (1) | A4_a (2) |  |  | A4_a (1) |  |  |  |  |
|  |  |  |  |  | A4_STP1 (1) |  |  |  |  |  |  |  |
|  |  |  |  |  | A66_STP1 (1) |  |  |  |  |  |  |  |
| AFR07 |  | A4_SEN1 (1) |  |  |  |  |  |  |  |  |  |  |
|  |  | A4_SEN2 (1) |  |  |  |  |  |  |  |  |  |  |
| AFR08* |  |  |  |  |  | A1_GAB1 (2) |  |  |  | A4_b (1) |  | A1_a (1) |
|  |  |  |  |  |  |  |  |  |  |  |  | A66_ZAF1 (1) |
| AFR09 |  |  |  |  |  |  |  |  |  |  |  |  |
| AFR10* |  |  |  |  |  |  |  |  |  |  |  |  |
| AFR11 | Z7_EGY1 (1) |  |  |  |  |  |  |  |  |  |  |  |
| AFR12* |  |  |  |  |  |  |  |  |  |  |  |  |
| AFR13* |  | A1_MAD3 (2) |  |  |  |  |  |  | A1_MAD3 (2) |  |  |  |
| EUR01* |  |  |  |  |  |  |  |  |  |  |  |  |
| EUR02* |  |  |  |  |  |  |  |  |  |  |  |  |
| EUR03 |  |  |  |  |  |  |  |  |  |  |  |  |
| EUR04* |  |  |  |  |  |  |  |  |  |  |  |  |
| EUR05* |  |  |  |  |  |  |  |  |  |  |  |  |
| EUR06* |  |  |  |  |  |  |  |  |  |  |  |  |
| EUR07* |  |  |  |  |  |  |  |  |  |  |  |  |
| EUR08* |  |  |  |  |  |  |  |  |  |  |  |  |
| EUR09 |  |  |  |  |  |  |  |  |  |  |  |  |
| EUR10* |  |  |  |  |  |  |  |  |  |  |  |  |
| EUR11* |  |  |  |  |  |  |  |  |  |  |  |  |
| EUR12* |  |  |  |  |  |  |  |  |  |  |  |  |
| EUR13* |  |  |  |  |  |  |  |  |  |  |  |  |
| EUR14 |  |  |  |  |  |  |  |  |  |  |  |  |
| EUR15 |  |  |  |  |  |  |  |  |  |  |  |  |
| EUR16* |  |  |  |  |  |  |  |  |  |  |  |  |

**Table S4c Distribution and occurrence of the 34 haplotypes of the partial ND2 gene for each European country.**

| ND2 ST | Portugal | Spain | France | Germany | Switzerland | Italy | Greece |
| --- | --- | --- | --- | --- | --- | --- | --- |
| SWIO01* |  |  |  |  |  |  |  |
| SWIO02* |  |  |  |  |  |  |  |
| SWIO03* |  |  |  |  |  |  |  |
| SWIO04* |  |  |  |  |  |  |  |
| SWIO05* |  |  |  |  |  |  |  |
| AFR01 |  |  |  |  |  |  |  |
| AFR02* |  |  |  |  |  |  |  |
| AFR03* |  |  |  |  |  |  |  |
| AFR04* |  |  |  |  |  |  |  |
| AFR05 |  |  |  |  |  |  |  |
| AFR06 |  |  |  |  |  |  |  |
| AFR07 |  |  |  |  |  |  |  |
| AFR08* |  |  |  |  |  |  |  |
| AFR09 | A11_PRT2 (1) |  |  |  |  |  |  |
|  | A14_PRT1 (1) |  |  |  |  |  |  |
|  | A16_PRT1 (1) |  |  |  |  |  |  |
| AFR10* | A1_b (1) |  |  |  |  |  |  |
| AFR11 |  |  |  |  |  |  |  |
| AFR12* |  |  |  |  |  |  |  |
| AFR13* |  |  |  |  |  |  |  |
| EUR01* |  | M7'_a (1) | M4_a (4) |  |  |  |  |
|  |  |  | M7'_a (2) |  |  |  |  |
| EUR02* |  |  | M6_FRA1 (2) |  |  |  |  |
| EUR03 |  |  | M4_b (2) |  |  |  |  |
| EUR04* |  |  |  |  |  | M3_a (1) |  |
|  |  |  |  |  |  | M7_a (1) |  |
|  |  |  |  |  |  | M7'_a (1) |  |
| EUR05* |  |  |  |  |  |  |  |
| EUR06* |  |  |  |  |  | M7_a (1) |  |
| EUR07* |  |  |  |  |  |  |  |
| EUR08* |  |  |  |  |  | C1_a (1) |  |
| EUR09 |  |  | C1_a (1) |  |  | C1_a (3) |  |
| EUR10* |  |  |  |  |  |  |  |
| EUR11* |  |  |  |  |  |  |  |
| EUR12* |  |  | C2_b (1) | C2_b (1) |  |  |  |
| EUR13* |  |  |  |  |  |  |  |
| EUR14 |  |  |  | C2_c (1) |  |  |  |
| EUR15 |  |  | C1_a (1) |  | C1_a (1) |  | C2_d (2) |
| EUR16* |  |  |  | C2_DEU1 (1) |  |  |  |
